# Catalyst‐Free Extraction of U(VI) in Solution by Tribocatalysis

**DOI:** 10.1002/advs.202404397

**Published:** 2024-07-01

**Authors:** Shuo Zhang, Feixue Gao, Ming Fang, Baoyi Liu, Bin Zhang, Zijian Zhong, Long Yu, Yifeng Zhang, Xiaoli Tan, Xiangke Wang

**Affiliations:** ^1^ College of Environmental Science and Engineering North China Electric Power University Beijing 102206 P. R. China; ^2^ School of Materials Science and Engineering Yan Shan University Qinhuangdao 066004 P. R. China; ^3^ School of Environmental Science and Engineering Guangdong University of Petrochemical Technology Maoming 525000 P. R. China

**Keywords:** catalysis, extraction, H_2_O_2_, tribocatalysis, uranium

## Abstract

Extraction of U(VI) in water is of great significance in energy and environmental fields. However, the traditional methods usually fail due to the indispensable extra addition of catalyst, adsorbent, precipitant, or sacrificial agents, which may lead to enhanced extraction costs and secondary pollution. Here, a new efficient uranium extraction strategy is proposed based on triboelectricity without adding a catalyst or other additives. It is found only under the friction between the microbubbles (generated under ultrasonication) and the water flow, that reactive oxygen species (ROS) can largely be generated, which thus contributes to the solidification of U(VI) from water. In addition, the magnetic field can affect the phase of the product. Under mechanical stirring, the product contains (UO_2_)O_2_·2H_2_O, while which contains UO_2_(OH)_2_ and (UO_2_)O_2_·4H_2_O under the magnetic stirring. Quenching experiments are also carried out to explore the influence of environmental factors. Most importantly, it shows great potential in the extraction of U(VI) from seawater. This work proposes a catalyst‐free and light‐free strategy toward the solidification of U(VI) from water, which avoids the secondary pollution of the catalyst to the environment and is low‐cost, and has great potential in the real application.

## Graphical abstract

A technique for U(VI) solidification based on tri biocatalysis is performed without using a catalyst. A large amount of ROS is produced due to the friction between gas bubbles and water molecules, which thus solidifies U(VI) from water. This work paves a new way to extract U(VI) from wastewater and seawater.

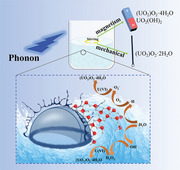

## 1 Introduction

Nuclear power is featured by high energy density and low carbon emissions, which is deemed as one of the most likely paths for the solution of energy shortage. In the nuclear industry, the most important element is uranium. The development of nuclear energy inevitably leads to the problems of resource shortage and environmental pollution. The reserve of uranium in the sea is ≈500–1000 times that on land, making the investigation of the extraction of uranium (U(VI)) from seawater a hot topic. Also, uranium is radioactive and highly toxic, once the uranium‐containing wastewater is released into the environment, it can cause significant harm to organisms.^[^ ^1^, ^2^, ^3^ ^]^ The common characteristic in uranium extraction from seawater and treatment of uranium‐containing wastewater is how to solidify soluble uranium (U(VI)) from water. For this purpose, various methods are developed, including adsorption, photocatalysis, reduction by Fe0, etc.,^[^ ^4^ ^]^ which have shown great application potential. A summary of some common methods is given in **Table** **1**.

**Table 1:** The advantages and disadvantages of common methods.

Methods	Advantages	Disadvantages
Ion exchange process	High‐efficiency, low‐cost	Secondary pollution, replace materials regularly
Chemical precipitation method	Simple craft, lowcost	Difficult separation of solid and liquid, secondary pollution
Membrane separation method	High purification coefficient; low energy consumption; flexible operation	Unstable performance
Biological method	Lowcost, no secondary pollution	Inefficiency, low recovery
Adsorption method	High purification coefficient, simple craft	Susceptible to co‐existing ions in water
Photocatalysis	High purification coefficient, wide application	Limited by the light source
Tribocatalysis	No secondary pollution, not easily affected by the environment	

Among the reported methods, the various catalytic methods have recently gained great concern due to their highly efficient and low cost, for example, photocatalysis and piezocatalysis.^[^ ^5^, ^6^, ^7^ ^]^ These two techniques both rely on the separation of electrons and holes in a catalyst under the irradiation of light or vibration (ultrasonication in a lab),^[^ ^2b^ ^]^ which could then directly or indirectly react with U(VI) in aqueous solution. However, various problems are inevitable while these techniques are applied due to the essential catalyst, for example, the sophisticated fabrication process and secondary pollution, thus leading to high costs either in the utilization or in the post‐treatment. Therefore, it is of great application potential to develop methods without using a catalyst to extract U(VI) from water. The ROS, for example, H_2_O_2_, ·O_2_^−^,^[^ ^8^ ^]^ etc., has been identified the important in solidification of U(VI) in photocatalytic or piezocatalytic processes.^[^ ^9^ ^]^

Actually, the ROS could also be found in some natural processes, for example, triboelectricity,^[^ ^10^ ^]^ which has been found for thousands of years.^[^ ^11^, ^12^ ^]^ Recently, based on the triboelectric phenomenon, researchers have developed a new catalytic way of tribocatalysis utilizing the separated electrons and holes at the solid interface to react with pollutant or water molecules. As far as we know, only dozens of publications are related to tribocatalysis, and mainly focused on the elimination of organic pollutants,^[^ ^8^ ^]^ and most of them ascribe the catalysis to the charge separation based on the frictional electricity between the interface of two solid matters, which makes the catalyst inevitable. These works present the possibility of the generation and evolution of radicals based on triboelectrification. However, the triboelectric phenomenon could be achieved at the interface more than solid–solid, but also solid–liquid, liquid–liquid, liquid–gas, and gas–gas. The friction of the inner matter in a catalytic system to separate charges may make the catalysis more environmentally friendly and economical.

Enlightened by the aforementioned discussion, it is very possible and important to implement a novel technique to solidify U(VI) without a catalyst. Therefore, in this work, we first propose a technique to solidify U(VI) from water by tribocatalysis, without introducing a solid catalyst. The mechanism the tribocatalysis in removing/extracting U(VI) in water is systematically investigated. This work presents a new facile and low‐cost route to separate U(VI) from water solutions, and the secondary pollution of a catalyst to the environment could be avoided, which may propose more applications in this field.

## 2 Results and Discussion

### 2.1 The Characterizations of the Catalytic Products

**Figure 1:**
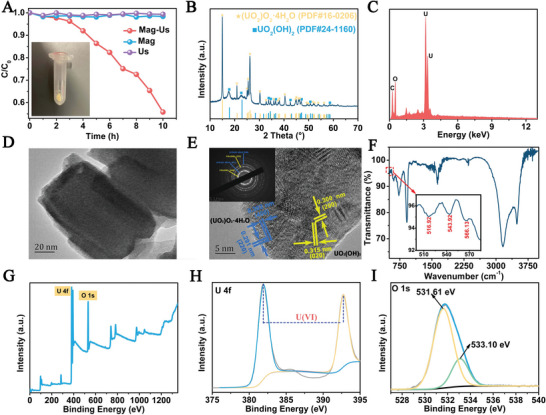
A) The tribocatalytic curve of U(VI) without a catalyst (“Mag” stands for magnetic stirring, “Us” stands for ultrasonication); XRD B), EDS C), TEM D), HRTEM E), SAED (the inset of Figure 1E), FTIR F) patterns, XPS survey G), high‐resolution XPS spectra of U 4f H) and O 1s I) of the obtained products.

The extraction of U(VI) from water has a great impact on the energy and environmental fields. Recently, to pursue more practical methods, different extraction methods have been developed. Among them, the piezocatalysis under ultrasonication has aroused great interest,^[^ ^5^, ^7^ ^]^ and is still inevitable to utilize catalysts that may induce secondary pollution and increase the application cost due to reliance on sophisticated fabrication equipment. To lower the extraction/separation cost, and avoid secondary pollution, this work presents a strategy without utilizing a catalyst. In this work, it is unexpected that the U(VI) concentration in water decreased under the synergistic effect of ultrasonication and magnetic stirring, which is shown in **Figures** **1**A and S1 (Supporting Information) (pH 4.4, 120 W, 40 kHz, magnetic stirring with rotation speed of 2680 rpm). However, the concentration of U(VI) remained unchanged without ultrasonication or without magnetically stirring. Under the coaction of ultrasonication and magnetically stirring, the concentration of U(VI) in the solution declined slowly in the first 3 h, then became fast until 10 h, and some yellow precipitations could be observed with the naked eye (the inset in Figure 1A). This phenomenon has not been reported by other researchers as far as we know, which does not rely on the adsorbents and catalysts, and does not need any sacrificial agents and irradiation of light.^[^ ^2^, ^13^ ^]^ There are no relevant publications on the removal/extraction of metal ions directly through ultrasonication, let alone U(VI) in water. Under such a scenario, the separation of U(VI) in solution only under mechanical vibration (sonication/stirring) makes the process lower cost and more available in the application.

Due to the novelty of this method, it is important to disclose the mechanism contributing to the solidification of U(VI) under ultrasonication and magnetic stirring. Then the product is collected and analyzed by XRD (Figure 1B), from which the XRD peaks of the product could be well indexed to uranyl peroxide (UO_2_)O_2_·4H_2_O, JCPDS No. 160 206) and uranyl hydroxide (UO_2_(OH)_2_, JCPDS No. 241 160). Only the U and O elements could be observed in the EDS spectrum of Figure 1C. UV–vis absorption experiment is carried out on the solution before sonication (0 h) and the dispersion after 10 h sonication. It could be seen no obvious absorption peak is observed at 350 nm before ultrasonication (the green line), while for the sample after sonication for 10 h, an absorption peak is found at ≈350 nm (Figure S2A, Supporting Information), which could be ascribed to the formation of (UO_2_)O_2_·4H_2_O and UO_2_(OH)_2_ solid complexes^[^ ^14^ ^]^ and further confirm the solidification process of U(VI) after sonication. The morphology of the obtained precipitates was characterized by a FESEM image (Figure S2B, Supporting Information), where the sample presents some rectangular morphology. Further investigation by TEM (Figure 1D; Figure S2C, Supporting Information) confirms the nanosheet structure, with a length of ≈150 nm and a width of 70 nm. More importantly, the lattice spacings of 0.300 and 0.315 nm in the HRTEM image (Figure 1E) correspond to the (200) and (020) crystal planes of UO_2_(OH)_2_, and the lattice spacings of 0.302 and 0.291 nm correspond to (400) and (220) crystal planes of (UO_2_)O_2_·4H_2_O, respectively. The corresponding SAED (Figure 1E) pattern further indicates some spot‐rings due to the large quantities of the nanocrystals contained in the view of the electron spots. Also, the co‐existed spots corresponding to peroxide and hydroxide confirm the co‐deposition of them. Further investigation is performed by the FTIR spectrum (Figure 1F), from which the absorption band at 607.55 cm^−1^ corresponds to the asymmetric tensile vibration of the U─O bond.^[^ ^15^ ^]^ The absorption bands at 727.16 and 912.26 cm^−1^ correspond to the symmetric and asymmetric stretching vibrations of the U═O bond, respectively. The absorption band at 1629.23 cm^−1^ corresponds to the bending vibration of ─OH in H_2_O, while the strong absorption bands at 3161.58 and 3486.78 cm^−1^ correspond to the O─H─O symmetric stretching and O─H stretching vibrations, respectively. In addition, two weak absorption bands at 566.13 and 516.92 cm^−1^ could be ascribed to the symmetric and antisymmetric stretching vibrations of U─OH, respectively, and the absorption band at 543.92 cm^−1^ is also associated with the U─O─H bond in UO_2_(OH)_2_.^[^ ^16^ ^]^ The strongest peak in the Raman spectrum (Figure S2D, Supporting Information) at 818.54 cm^−1^ corresponds to a symmetric stretching vibration of the O═U═O bond. The peak at 866.49 cm^−1^ in the Raman spectrum corresponds to the peroxide stretching of the O─O bond to bridge the oxygen ligand.^[^ ^17^ ^]^

To further confirm the above conclusions, an XPS test is carried out and the results are shown in Figure 1. From the survey spectrum (Figure 1G), U and O are clearly observed. The high‐resolution spectrum (Figure 1H) shows double peaks at 381.89 and 392.73 eV corresponding to U(VI), which proves the presence of U(VI) once again. The O 1s spectrum (Figure 1I) shows the overlap of the two peaks. The peak with a lower binding energy (531.61 eV) corresponds to O^2−^. Another peak with a higher binding energy (533.10 eV) corresponds to either hydroxide (OH^−^) or peroxide (O_2_^2−^).^[^ ^18^ ^]^ This allocation is reasonable because peroxides should exhibit a higher O 1s binding energy than oxides which have a formal charge of O^2−^.^[^ ^19^ ^]^ These results all confirmed the existence of UO_2_(OH)_2_ and (UO_2_)O_2_·4H_2_O in the products.

### 2.2 Affecting Factors on the Catalysis of U(VI)

Generally, for a catalytic process, there are always lots of factors that may influence the reaction. In this work, the effect of reactant concentration on catalysis is investigated. It can be found that the degradation rate follows 30 > 10 > 50 > 100 > 70 ppm (Figure S3, Supporting Information), and all the curves still tend to decline at 10 h indicating the solidification of U(VI) by catalysis is a highly efficient process. Also, the removal rate of U(VI) in 30 ppm solution is the fastest in 10 h. At U(VI) concentration <30 ppm, the catalytic efficiency increased as increasing the concentration. However, the extra high initial concentration of U(VI) prolongs the catalytic time to achieve complete removal due to insufficient reactants. The effect of the stirring speed of the magnetic field on the extraction/removal of U(VI) is also investigated (Figure S4, Supporting Information). It is easy to find that when the stirring speed is 1370 rpm, the concentration of the U(VI) solution is hardly changed. As the stirring speed achieves 2140 rpm, the U(VI) concentration in the solution begins to decline, and the higher the stirring speed, the faster the catalytic rate. In addition, there seems to always exist a “plateau” in the catalytic curve, and the increase in rotational speed can shorten the “plateau” of the reaction, that is the catalytic reaction at the stirring speed of 2680 rpm obviously starts at around the 5th hour, while at the stirring speed is 3660 rpm, the catalytic reaction starts at the 2nd hour. This may be because the low rotational speed cannot achieve the generation energy of ROS necessary for catalytic reactions. Only when the ROS generation energy threshold is reached can ROS be generated and then participate in the catalytic reaction? Nonetheless, although such a high speed of 3660 rpm is a favor for catalysis, the temperature of the motor is so high that could affect the service life of the equipment. Therefore, the following experiments are implemented at the magnetic stirring speed of 2680 rpm.

**Figure 2:**
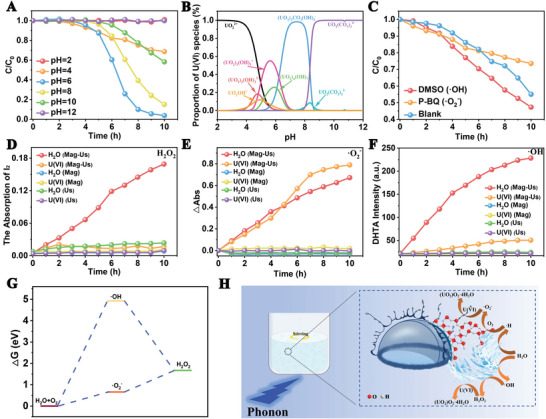
A) The curve of U(VI) concentration over time at different pH values; B) the pH‐dependent speciation of U(VI) in solution (initial U(VI) = 50 mg·L^−1^, T = 293 K); C) the catalysis experiment with different sacrificial agents; the curves of absorption variation versus time for I_2_ D), ·O^2−^ E); the curves of intensity variation versus time for dihydroxyterephthalic acid (DHTA) F); G) reaction free energy diagram of ROS; H) schematic diagram of the tribocatalytic extraction of U(VI). (“Mag” stands for magnetic stirring; “Us” stands for ultrasonication; H_2_O_2_ is determined by the iodization method, △Abs = Absinitial – Absx, x = reaction time.).

The effect of pH value on catalytic performance is shown in **Figure** **2**A. No obvious decrease is observed at pH 2 (Figure S5A, Supporting Information). As increasing the pH value, the removal rate of U(VI) increased gradually and achieved the highest at pH 6 (Figure S5C, Supporting Information). After that, a further increase in the pH value may decrease the removal rate, and lose the ability at pH 12 (Figure S5F, Supporting Information). To comprehensively discuss the reasons contributing to the pH‐dependent catalysis, two aspects should be considered, the species of U(VI) in the solution and the solidification agents for U(VI) at different pH values. The U(VI) species in solution at different pH values are simulated by Visual MINTEQ ver.·3.1 (Figure 2B). From the Figure, at pH 2.0, the dominant U(VI) species is UO_2_^2+^, and as the pH increased over 3.0, the ratio of UO_2_^2+^ gradually decreased, and then decreased fast over pH 4.0. At the same time, some OH^−^‐containing hydrated ions occurred and achieved the maximum quantity at different pH. As the pH value increased over 5.0, (UO_2_)_2_CO_3_(OH)^−^ occurred and became the dominant one at pH >7.0, and maintained constant until pH 8.0. After that, UO_2_(CO_3_)_3_^4−^ occurred and dominated in the solution at a pH >9.0. Considering the result of Figure 2A, the most likely catalyzed species is (UO_2_)_x_(OH^−^)_y_ (where x = 1, 2, 3, and 4, y = 1, 2, 5, and 7).

### 2.3 Catalytic Mechanism

It should be noted that from Figure 1A, no U(VI) could be extracted/removed only under ultrasonication or magnetic stirring. The extraction of U(VI) could only be achieved under the synergistic effect of ultrasonication and magnetic stirring, indicating their indispensable performances in catalysis. Due to the heat released in the process of ultrasound, in order to eliminate this effect, the entire experiment is carried out under the condition of 20 °C controlled by a circulating thermostatic water tank. However, understanding the specific roles of the two aspects is of great value. Therefore, this part has been further studied. Generally, as well known, large quantities of air bubbles may be generated due to the cavitation effect under ultrasonication. As stirring works, the bubbles rub with water molecules. In this condition, as long as the stirring velocity is fast enough, the bonds may break to generate radicals due to the rub and bubbles break.^[^ ^20^ ^]^ Thus, the generation of radicals and the extraction of U(VI) could be ascribed to the tribocatalysis. It should be noted that this work is different from the reported mechanism of triboelectric plasma,^[^ ^11^, ^12^ ^]^ where the friction pair is utilized to generate an electric current to induce a discharge ionization effect. However, all the applications derived from triboelectric phenomena prove that this field has good prospects for development.

Therefore, radical scavengers are added to determine their roles in the catalytic process. DMSO and P‐BQ are added to the U(VI)‐containing solutions as the capture agents of ·OH, and ·O_2_^−^, respectively. From Figure 2C, the addition of DMSO obviously promotes the catalytic reaction, while the addition of P‐BQ plays a promotion effect in 6 h and a counteraction after that.

However, it is still hard to build the evolution route from U(VI) to UO_2_O_2_·4H_2_O and UO_2_(OH)_2_. Generally, in addition to the common radicals of ·O_2_^−^ and ·OH in water, H_2_O_2_ is another important ROS that usually exists in catalytic circumstances, for example, photocatalysis and piezocatalysis,^[^ ^5^, ^21^ ^]^ which could be produced through the evolution of ·O_2_^−^ and ·OH. To verify whether there exists H_2_O_2_ in the catalysis, the generations of H_2_O_2_ in different conditions are determined (Figure 2D). It should be noted that if only under magnetic stirring or ultrasonication, almost no H_2_O_2_ could be detected whether in water or in that containing U(VI) (except a very small amount of H_2_O_2_ could be observed in pure water under ultrasonication). However, under the effect of magnetic stirring and ultrasonication in water, the concentration of H_2_O_2_ (proportional to the absorption of I_2_) increases significantly, while the concentration of H_2_O_2_ decreases to a hardly negligible level with further adding U(VI). This implies that the H_2_O_2_ may contribute to the formation of peroxide and be consumed.

From the abovementioned results, the ·OH and ·O_2_^−^ play an important role in the U(VI) catalysis, and finally produced UO_2_O_2_·4H_2_O and UO_2_(OH)_2_. The radicals are usually transient products in a catalytic process, which means these two radicals should be responsible for the formation of the stable H_2_O_2_. Therefore, the generations of ·OH and ·O_2_^−^ are tested and shown in Figure 2E,F. It is interesting to find that both of them are at a low level only under magnetic stirring or ultrasonication. At the coeffect of magnetic stirring and ultrasonication, the concentrations of them increase greatly in pure water. However, in the U(VI)‐containing system, the concentration of ·O_2_^−^ (proportional to the difference of the adsorption of NBT) maintains the same level compared to that in pure water at the first ≈4 h, then increases and exceeds that in pure water. This process basically corresponds to the slow stage and fast stage of U(VI) solidification in Figure 1A, indicating the solidification of U(VI) is a process of generating ·O_2_^−^ (Equation 1).

(1) $$U\left(\mathrm{VI}\right)+H_{2}O_{2}+4H_{2}O\to \left(UO_{2}\right)O_{2}\cdot 4H_{2}O+\cdot {O_{2}}^{-}+H^{+}$$

As for ·OH, its concentration in the system containing U(VI) is far below that in pure water, indicating the solidification of U(VI) is a process consuming ·OH, which may transfer to H_2_O_2_ according to Equation (2):

(2) $$2\cdot \mathrm{OH}↔H_{2}O_{2}$$

Based on the above analysis, equations related to the solidification of U(VI) were speculated and written as follows:^[^ ^14^, ^15^, ^22^ ^]^

(3) $$U\left(\mathrm{VI}\right)+2OH^{-}↔UO_{2}{\left(\mathrm{OH}\right)}_{2}$$

(4) $$UO_{2}{\left(\mathrm{OH}\right)}_{2}+H_{2}O_{2}+2H_{2}O↔\left(UO_{2}\right)O_{2}\cdot 4H_{2}O$$

(5) $$U\left(\mathrm{VI}\right)+H_{2}O_{2}+4H_{2}O↔\left(UO_{2}\right)O_{2}\cdot 4H_{2}O+2H^{+}$$

In solution, U(VI) can react directly with OH^−^ to form UO_2_(OH)_2_ (Equation 3). UO_2_(OH)_2_ can further react with the generated H_2_O_2_ to form (UO_2_)O_2_·4H_2_O (Equation 4), and part of U(VI) can directly react with H_2_O_2_ to generate (UO_2_)O_2_·4H_2_O (Equation 5). The Gibbs free energies of·OH and ·O_2_^−^ are calculated to demonstrate their potential to transform into H_2_O_2_, which could be deduced from Figure 2G and Table 1. In addition, the reactions of UO_2_(OH)_2_ and U(VI) with H_2_O_2_ are both acidifying processes and then could be indirectly proved by testing the pH variations before and after catalysis. From Figure S10 (Supporting Information), the pH value decreased after the reaction, which means the reaction is a H^+^‐releasing process.

The above experiments demonstrate the evolution process of ROS and their effects in the U(VI) solidification under the coeffect of magnetic stirring and ultrasonication. However, a question here is how the magnetic stirring and the ultrasonication affect the evolution of ROS. It is well admitted that ROS could be generated during the break of the microbubbles, while their yields are very small in our experiments unless magnetic stirring is implemented. This is also proved by electrochemical experiments (Figure S11, Supporting Information). The current response is the largest simultaneously under stirring and ultrasound. Actually, several researchers have found the important role of stirring in a catalytic process. In some recent publications, the stirring was considered to induce different velocities between solid catalysts and organic pollutants,^[^ ^19^, ^23^ ^]^ most of which are achieved by the triboelectric effect between solid catalysts (i.e., bismuth oxyiodate, barium strontium titanate) and polytetrafluoroethylene. However, in our experiments, no solid catalyst is added to the system, then which mechanism should be responsible for the catalysis? On the occasion of ultrasonication alone, there is no relative movement between bubbles and water. In the occasion of magnetic stirring alone, there is no probable interface for triboelectrification. Therefore, the U(VI) catalytic process could only happen under the coeffect of magnetic stirring and ultrasonication.

Considering the facts that bubbles are generated during sonification at the same time, and the magnetic stirring induces different velocities of solution at different positions, a new mechanism could be proposed: the magnetic stirring moves the bubbles in the solution, which may then undergo friction with water molecules, in other words, the friction happens between the liquid and gas interface. The surface layer of the bubbles can be constrained as a thin layer of water molecules, which are in a non‐equilibrium state due to the effect of surface tension. Then the adjacent water molecules may be strongly captured by the bubble surface layer, however, under the stirring, it may also undergo the pulling force by the molecules in the distance. Thus, the molecules may be snapped due to the two contrary forces Equation (6):

(6) $$H_{2}O↔\cdot \mathrm{OH}+\cdot H$$

The generated ·H is not stable, which could then react with the soluble oxygen to form ·O_2_^−^ according to Equation (7):

(7) $$O_{2}+\cdot H↔H^{+}+\cdot {O_{2}}^{-}$$

After that, the generated radicals could then react/transform to the other radicals according to the previous Equations ((1), (2), (3), (4), (5)) to solidify U(VI), which can be vividly shown in Figure 2H.

**Figure 3:**
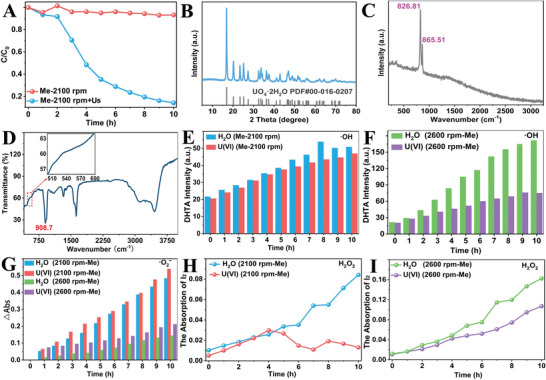
A) The effect without magnetic field in the tribocatalysis of U(VI); B–D), the XRD, Raman, and FTIR patterns of the product under 2100 rpm mechanical stirring and ultrasound; E) the curves of intensity variation versus time for DHTA under ultrasonication and 2100 rpm mechanical stirring speed in ultrapure water and U(VI) solution; F) the curves of intensity variation versus time for DHTA under ultrasonication and 2600 rpm mechanical stirring in ultrapure water and U(VI) solution; G) the curves of absorption variation versus time for ·O^2−^ under ultrasonication and different mechanical stirring speeds in ultrapure water and U(VI) solution; H) the curves of absorption variation versus time for I_2_ under ultrasonication and 2100 rpm mechanical stirring speed in ultrapure water and U(VI) solution; I) the curves of absorption variation versus time for I_2_ under ultrasonication and 2600 rpm mechanical stirring speed in ultrapure water and U(VI) solution. (“Me” stands for mechanical stirring, “Us” stands for ultrasonication).

It should be noted that another commonly used stirring manner is mechanical stirring, which is different from magnetic stirring by lack of an extra magnetic field. The important effect of magnetic field on catalysis has been confirmed in previous studies.^[^ ^7c^ ^]^ Therefore, to explore the magnetic field on the tribocatalysis of U(VI), similar experiments based on mechanical stirring are implemented. 50 ppm uranyl solution is used, the temperature of the whole experiment is controlled at 20 °C, and the pH value of the solution is adjusted to 4.4. From **Figures** **3**A and S12 (Supporting Information), the U(VI) concentration in the solution decreased as the ultrasonication time, indicating that the extraction of U(VI) could also be achieved under the synergetic effect of ultrasound vibration and mechanical stirring. Also, no U(VI) could be catalyzed only under mechanical stirring. From Figure S13 (Supporting Information), the best performance under mechanical stirring and ultrasonication is as the stirring speeds achieved at 2100 rpm, and that under 2600 rpm is a little worse. Then the following experiment is performed at 2100 rpm mechanical stirring.

To investigate the mechanism contributing to the extraction of U(VI) under mechanical stirring, the product collected under 2100 rpm stirring is characterized. The FESEM shows the product shows nanosheet‐like morphology, which is ≈150–200 nm in diameter and 10 – 20 nm in thickness (Figure S14, Supporting Information). From Figure 3B, it is interesting to find that the product only contains (UO_2_)O_2_·2H_2_O (JCPDS No. 000 160207), which is different from that obtained by magnetic stirring. In addition, the Raman spectrum (Figure 3C) indicates two peaks, the peak at 826.81 cm^−1^ corresponds to the symmetric stretching vibration of the O═U═O bond, and the peak at 865.51 cm^−1^ corresponds to the peroxide stretching vibration of the O─O bond.^[^ ^17^ ^]^ The absorption band at 908.70 cm^−1^ in the FTIR spectrum (Figure 3D) corresponding to the asymmetric tensile vibration of the U═O bond further verifies the existence of the U(VI) containing product. Notably, there is no significant absorption band between 500 and 600 cm^−1^, demonstrating the absence of UO_2_(OH)_2_, which is consistent with XRD results.

From the above experiments, the product ((UO_2_)O_2_·2H_2_O) obtained by mechanical stirring and ultrasonication is different from those by magnetically stirring ((UO_2_)O_2_·2H_2_O + UO_2_(OH)_2_), which means the solidification mechanism is different. Accordingly, the generation and evolution of the radicals are investigated, which is shown in Figure 3. The quantity of the ·OH radicals in water without U(VI) is basically equal to that with U(VI) (Figure 3E; Figure S15A,B, Supporting Information), and both are at the same level as that under 2600 rpm (with U(VI), Figure 3F; Figure S15D, Supporting Information) stirring and magnetically stirring. However, it increases a lot under 2600 rpm mechanical stirring without U(VI) (Figure S15C, Supporting Information). This means the low rpm mechanical stirring (2100) generates small quantities of ·OH, which was then consumed by the tribocatalytic process of U(VI). However, from Figure 3G and Figure S16 (Supporting Information), the ·O_2_^−^ generated without magnetic fields only achieves 1/3–1/2 of that with magnetic fields (2600 rpm, by comparing to Figure 2E), while under 2100 rpm mechanical stirring, it reaches the same level of that under 2600 rpm magnetic stirring. This result indicates the magnetic field or mechanical stirring at low rpm is favorable for the generation of ·O_2_^−^. It should also be noted that the generated ·O_2_^−^ in solution containing U(VI) are much more than that in pure water either at 2100 rpm or at 2600 rpm, indicating the tribocatalysis of U(VI) is a process of releasing ·O_2_^−^. However, the generation of H_2_O_2_ in this work at 2100 rpm mechanical stirring decreased as U(VI) began to precipitate compared to that in water (Figure 3H; Figure S17A,B, Supporting Information), while it is still high at 2600 rpm mechanical stirring (Figure 3I; Figure S17C,D, Supporting Information). This means the low stirring speed is favorable for the peroxidation of U(VI) while the high stirring speed is favorable for the generation of H_2_O_2_.

Compared with the experiment under 2600 rpm mechanical stirring, the quantities of both ·OH and H_2_O_2_ produced in water and U(VI) solutions under the stirring speed of 2100 rpm are relatively small, but the amount of ·O_2_^−^ is relatively high. Then the reason for the low ·O_2_^−^ generation in aqueous solution with high stirring speed may be that extra high stirring speed reduces dissolved oxygen in the water and thus affects the generation of ·O_2_^−^. It is worth noting that the catalytic performance under the speed of 2100 rpm is much better than that under the speed of 2600 rpm. Therefore, H_2_O_2_ may not be necessary for the formation of (UO_2_)O_2_·2H_2_O, which is most likely to be produced directly by ·O_2_^−^ (Equation 8).

(8) $$U\left(\mathrm{VI}\right)+\cdot {O_{2}}^{-}\to \left(UO_{2}\right)O_{2}\cdot 2H_{2}O$$

However, how does the magnetic field affect the formation of the products? Generally, magnetic agitation mainly affects the active ROS in the solution. The introduction of an external magnetic field can optimize the spin‐polarized directions and energy of electrons. It has been reported that the applied magnetic field can enhance the electron spin parallel arrangement and inhibit the bonding between the antiparallel spin hydroxyl groups, thus improving the catalytic activity.^[^ ^24^ ^]^ In most catalytic reactions, ROS such as ^1^O_2_, ·O_2_^−^ and ·OH are the determining factors of catalytic performance. Therefore, magnetic fields can control the spin of these ROS, which can modulate their reactivities.^[^ ^25^ ^]^

**Figure 4:**
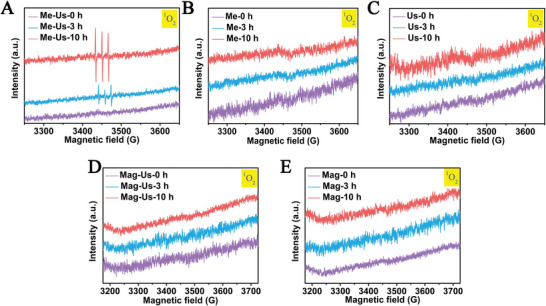
The EPR spectra in different times: The ESR spectra under mechanical stirring and ultrasonication A); the ESR spectra under mechanical stirring B); the ESR spectra under ultrasonication C); the ESR spectra under magnetic stirring and ultrasonication D); the ESR spectra under magnetic stirring E). (“Me” stands for mechanical stirring, “us” stands for ultrasonication, “Mag” stands for magnetic stirring).

The formation of reaction intermediates under different conditions is studied by electron paramagnetic resonance (EPR) (**Figure** **4**). It can be seen from the results that ^1^O_2_ is present only in the presence of both mechanical stirring and ultrasound. The ^1^O_2_ signal is detected 3 h after the reaction, but the signal is more pronounced 10 h after the reaction. This shows that ^1^O_2_ is gradually increasing as the reaction progresses. It is highly likely that the magnetic field affects the spin arrangement of the electrons and thus the presence of ROS in the solution. Similarly, the difference between the products of magnetic stirring and mechanical stirring may also be caused by this.

**Figure 5:**
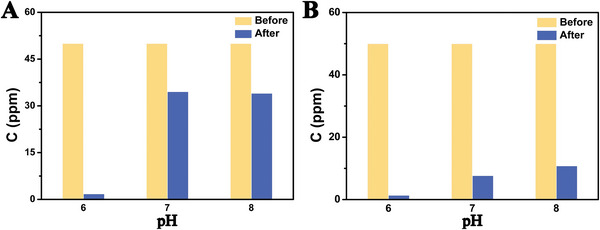
Catalytic experiments are carried out in seawater with different pH values (the catalytic reaction time is increased to 20 h): Catalytic experiments in the presence of magnetic stirring A); catalytic experiments in the presence of mechanical stirring B).

In addition, in order to verify the effect of practical application, some environmental factors (ultrasonic power, temperature, cation, and anion) are also tested (details see Figures S18–S21, Supporting Information). Due to the superior performance of this strategy, the U(VI) extraction in seawater is carried out. It can be seen from **Figure** **5**, that the U(VI) contents in seawater with different pH values all reduced. In particular, mechanical stirring has better catalytic performance than that under magnetic stirring. At pH 8 (the natural seawater), the concentration of U(VI) decreased by 80% after 20 h tribocatalysis, indicating a great application potential of this technique in the extraction of U(VI) from seawater.

## 3 Conclusion

In conclusion, a new technique for U(VI) solidification based on tribiocatalysis is confirmed without using a catalyst and avoids secondary pollution. The tribocatalysis is mainly due to the friction between the ultrasonication‐generated bubbles and water molecules, which then may generate lots of ROS for the solidification of U(VI). The product under magnetic stirring ((UO_2_)O_2_·4H_2_O and UO_2_(OH)_2_) is different from that under mechanical stirring ((UO_2_)O_2_·4H_2_O), which is proved to be due to the effect of the magnetic field on the generation of the ROS species. The solidification of U(VI) under magnetic stirring is due to the reaction with H_2_O_2_, ·OH, and ·O_2_^−^, while which is mainly due to ·O_2_^−^ under mechanical stirring. The green and environmental protection, highly efficient, and low‐cost performance of the U(VI) tribocatalytic strategy provides a new idea and strong application prospects in the treatment of U(VI) containing water, especially from seawater.

## Conflict of Interest

The authors declare no conflict of interest.

## Author Contributions

M.F. and X.T. designed the experiments. S.Z. performed the catalytic experiments. S.Z. and L.Y. performed the characterization. F.G. and B.L. were responsible for the preparation of the solution. B.Z. was responsible for the calculation of free energy. S.Z. and Z.Z. measured the concentration of U(VI) on a UV–vis absorption spectrophotometer. S.Z., F.G., Y.Z., and X.W. processed the experimental data. The manuscript was written by S.Z. and M.F. All authors participated in the discussion and analysis of the paper.

## Supporting information

Supporting Information

## Data Availability

The data that support the findings of this study are available from the corresponding author upon reasonable request.
