# Supplementary material for: Catalyst‐Free Extraction of U(VI) in Solution by Tribocatalysis
Source: Adv Sci (Weinh). 2024 Jul 1;11(33):2404397. doi: 10.1002/advs.202404397 (PMC11434018; doi:10.1002/advs.202404397)
Supplement: Supplementary file 1 — Supporting Information [file ADVS-11-2404397-s001.docx]

**Supporting Information**

**Catalyst-free Extraction of U(VI) in Solution by Tribocatalysis**

Shuo Zhang,^a^ Feixue Gao,^a^ Ming Fang,*^a^ Baoyi Liu,^a^ Bin Zhang,^b^ Zijian Zhong,^a^ Long Yu,^c^ Yifeng Zhang,^a,c^ Xiaoli Tan*^a^, Xiangke Wang^a^

[a] College of Environmental Science and Engineering, North China Electric Power University, Beijing 102206, P.R. China

E-mail: mfang@ncepu.edu.cn; xltan@ncepu.edu.cn

[b] School of Materials Science and Engineering, Yan Shan University, Qinhuangdao, 066004, P.R. China

[c] School of Environmental Science and Engineering, Guangdong University of Petrochemical Technology, Maoming 525000, China

**Contents**

[**Supporting Information** 1](#_Toc167729226)

[1. Experimental section 4](#_Toc167729227)

[1.1 Chemicals 4](#_Toc167729228)

[1.2 Catalytic experiment 4](#_Toc167729229)

[1.3 Determination of H_2_O_2_ 5](#_Toc167729230)

[1.4 Determination of ·O_2_^-^ 5](#_Toc167729231)

[1.5 Determination of ·OH 5](#_Toc167729232)

[1.6 Electrochemical experiments 5](#_Toc167729233)

[1.7 Statistical Analysis 6](#_Toc167729234)

[2. Characterizations 7](#_Toc167729235)

[3. Calculation Method 8](#_Toc167729236)

[4. Supplementary Figures 9](#_Toc167729237)

[Figure S1. 9](#_Toc167729238)

[Figure S2. 10](#_Toc167729239)

[Figure S3. 11](#_Toc167729240)

[Figure S4. 12](#_Toc167729241)

[Figure S5. 13](#_Toc167729242)

[Figure S6. 14](#_Toc167729243)

[Figure S7. 15](#_Toc167729244)

[Figure S8. 16](#_Toc167729245)

[Figure S9. 17](#_Toc167729246)

[Figure S10. 18](#_Toc167729247)

[Figure S11. 19](#_Toc167729248)

[Figure S12. 20](#_Toc167729249)

[Figure S13. 21](#_Toc167729250)

[Figure S14. 22](#_Toc167729251)

[Figure S15. 23](#_Toc167729252)

[Figure S16. 24](#_Toc167729253)

[Figure S17. 25](#_Toc167729254)

[Figure S18. 26](#_Toc167729255)

[Figure S19. 28](#_Toc167729256)

[Figure S20. 30](#_Toc167729257)

[Figure S21. 32](#_Toc167729258)

[5. Supplementary Table 33](#_Toc167729259)

[Table S1. 33](#_Toc167729260)

[6. References 34](#_Toc167729261)

# Experimental section

## 1.1 Chemicals

AlCl_3_ is purchased from Sinopharm Group Chemical Reagent Co. Ltd. UO_2_(NO_3_)_2_·6H_2_O, NaOH, HNO_3_, NaCl, CaCl_2_, Na_2_CO_3_, p-benzoquinone (P-BQ), dimethyl sulfoxide (DMSO) are purchased from Maclin Biochemical Co., Ltd (Shanghai, China). NaNO_3_ was purchased from West Asia Reagent Co., Ltd (Shandong, China). All the chemicals are analytically pure and can be used directly without further purification, and the solutions in the experiment were prepared with ultrapure water (18.25 MΩ cm). The seawater is obtained in Dalian, China.

## 1.2 Catalytic experiment

In a typical tribocatalysis experiment, 10 mL of a pre-configured 250 ppm uranyl solution is mixed with 40 mL of deionized water in a glass tube. With the aid of a magnetic/mechanical agitator, the tribocatalysis experiment is carried out by a self-made tribocatalysis reactor. Without special instructions, the experimental conditions are set at a temperature of 20 ℃, frequency of 40 kHz, power of 120 W and pH value of 4.4. The pH value of the reaction solution is adjusted by negligible diluted HNO_3_ and NaOH solutions. 1 mL uranyl reaction solution is taken out from the glass tube every 1 h, and then is rapidly filtered through a cellulose acetate syringe membrane (with an aperture of 0.22 μm). 2, 4-dinitrophenol, HNO_3_, buffer solution and Arsenazo III are added to the filtrate successively. After 10 h, the concentration of U(VI) in the solution is determined by ultraviolet-visible (UV-vis) spectrophotometry. In order to further study the application potential, NaCl (0.01 M, 0.05 M, 0.2 M), AlCl_3_ (0.01 M), CaCl_2_ (0.01 M) are used to study the influence of coexisting cations and ion concentration, and Na_2_CO_3_ (0.2 M), NaNO_3_ (0.2 M), NaCl (0.2 M) are used to study the effect of coexisting anions. In addition, ROS quenching experiments are carried out without changing the experimental conditions, except for extra adding DMSO and P-BQ to capture ·OH and ·O_2_^-^, respectively. In addition, a control experiment is conducted without ultrasonication.

## 1.3 Determination of H_2_O_2_

H_2_O_2_ is determined by the iodization method. Specifically, 1 mL reaction solution is taken every 1 h and filtered through the filter membrane. Then, 0.1 M KI and 0.01 M ammonium molybdate solutions are added to the filtrate and the volume is modulated to 25 mL by adding pure water. Then it is detected on UV-vis absorption spectrophotometer.

## 1.4 Determination of ·O_2_^-^

In the catalytic experiment, 5 mL H_2_O is replaced with 5 mL NBT (0.25×10^-3^ M), and then 4 mL reaction solution is taken out every 1 h. The solution is tested directly on a UV-vis absorption spectrophotometer after being filtered.

## 1.5 Determination of ·OH

The 25 mL H_2_O in the catalytic experiment is replaced with 25 mL terephthalic acid (1 mM), and then 4 mL reaction solution is taken out every 1 h. The solution is tested directly on a photoluminescence spectrometer after being filtered through the filter. Terephthalic acid reacts with •OH to form dihydroxyterephthalic acid. Therefore, the signal intensity of dihydroxyterephthalic acid detected by photoluminescence spectrometer, which indirectly reflects the formation of •OH.

## 1.6 Electrochemical experiments

The transient current response is performed on an electrochemical workstation by using a three-electorde system, in which 50 ppm U(VI) solution is used as electrolyte, an Ag/AgCl electrode as reference electrode and a Pt sheet as counter electrode.

## 1.7 Statistical Analysis

The absorbance of 5 ppm, 12.5 ppm, 25 ppm, 50 ppm and 125 ppm U(VI) solution prepared in advance is determined by UV spectrophotometry. The standard curve is obtained by fitting the concentration and the measured absorbance according to the linear relationship. In the whole catalytic experiment, the absorbance of U(VI) measured by UV spectrophotometry is converted into concentration according to the standard curve. “C_0_” is the concentration of U(VI) at reaction 0 h, and “C” is the concentration of U(VI) at any time. C/C_0_ is used to normalize the data.

# Characterizations

An X-ray powder diffractometer model SmartLab SE manufactured by Rigaku is used to record the X-ray diffraction pattern of the solid powder after the catalytic reaction. The infrared transmission spectra were obtained by adopting KBr lamination method on SHIMADZU-IRT racer-100. Raman spectrum is obtained by Accuman-SR510 portable Raman spectrometer (Marine Optics, Shanghai, China). The elemental composition and valence states of the collected materials are characterized by X-ray photoelectron spectroscopy (XPS, Thermo Fisher, ESCALAB 250Xi, USA, Al Kα (hν= 1486.7 eV)). Transmission electron microscopy (TEM, JEOL JEM-2010, 200 kV) and high-resolution transmission electron microscopy (HRTEM, JEOL JEM-2010, 200 kV) are obtained to study the morphology, size and lattice structure of the collected products. The photoluminescence (PL) spectra excited by 310 nm wavelength light are recorded by Perkin Elmer LS 55 fluorescence spectrophotometer at room temperature. The concentration of U(VI) is measured on a UV-vis absorption spectrophotometer (Shimadzu, UV-2700, Japan). The electron paramagnetic resonance (EPR) of the samples is measured with Brook microESR. The electrochemical experiments are obtained by the electrochemical workstation model CHI750e (Shanghai Chenhua Instrument Co., Ltd).

# Calculation Method

The Gibbs free energy of each substance is calculated by density functional theory (DFT). Three active substances (·OH, ·O_2_^-^ and H_2_O_2_) that may be formed during the reaction are considered. Since no catalyst is added in the catalytic experiment, the reaction intermediates play an important role in it. First, the heat of combustion of each substance is calculated, and the result is subtracted to get △H. Then, according to the entropy of each substance, the free energy can be calculated as follows:

△G = △H - T△S (1)

where △H is the heat of combustion calculated by DFT and △S is the change of entropy.

# Supplementary Figures


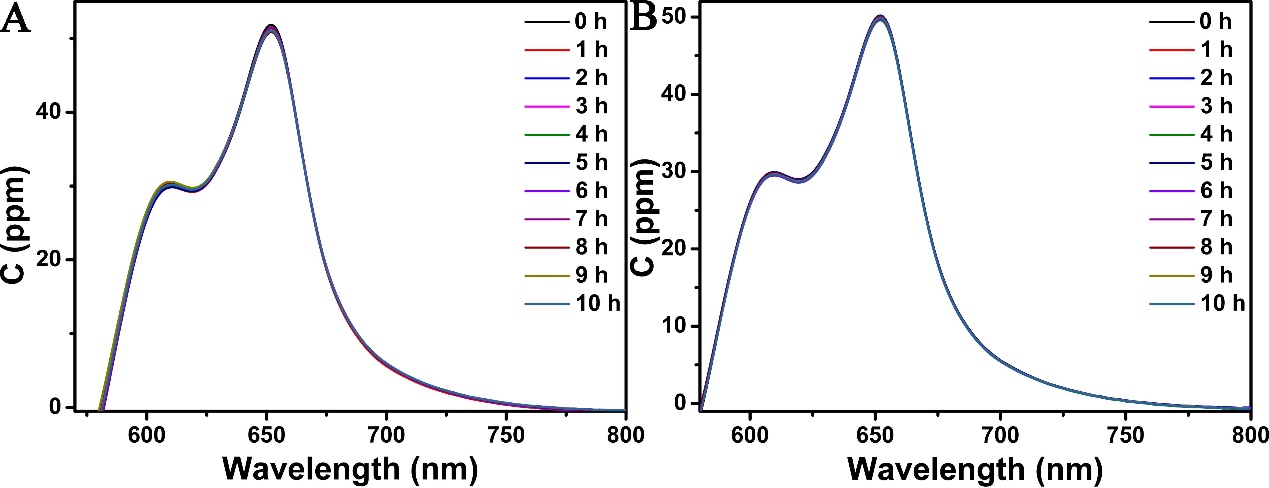


Figure S1. The curve of U(VI) concentration with time under magnetic stirring(A) and ultrasound(B), respectively.


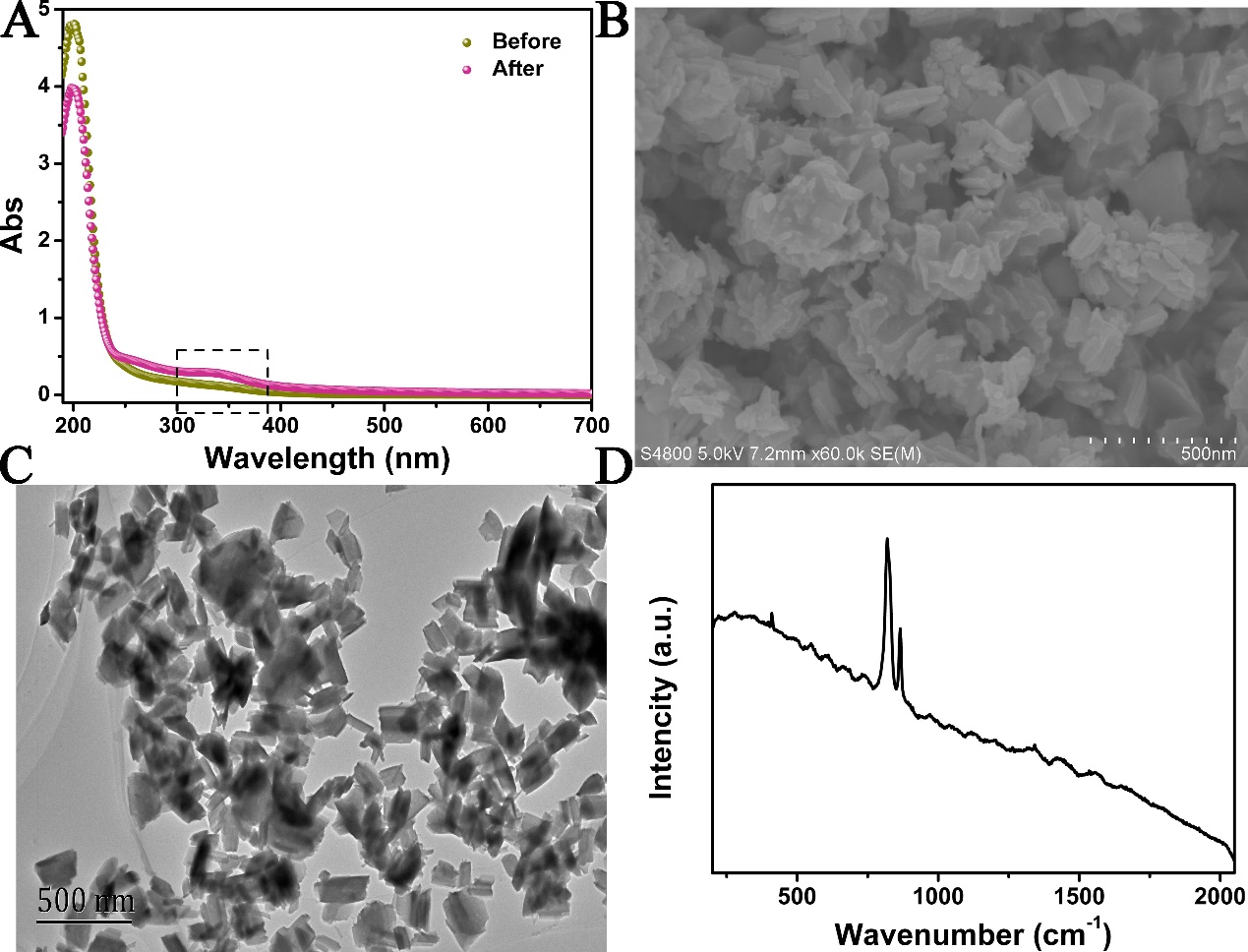


Figure S2. The UV-vis absorption spectra of solution before and after catalysis(A); SEM(B), TEM(C) and Raman(D) patterns of the sample.

.


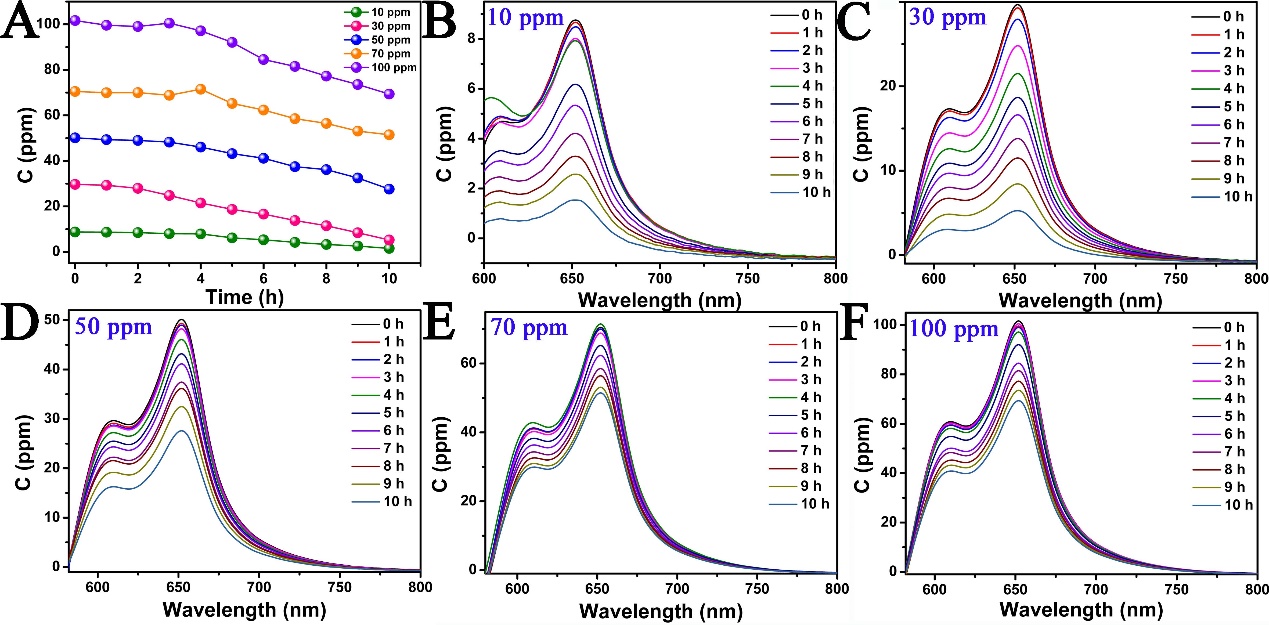


Figure S3. The concentration curve with time of U(VI) solution with different initial concentration.


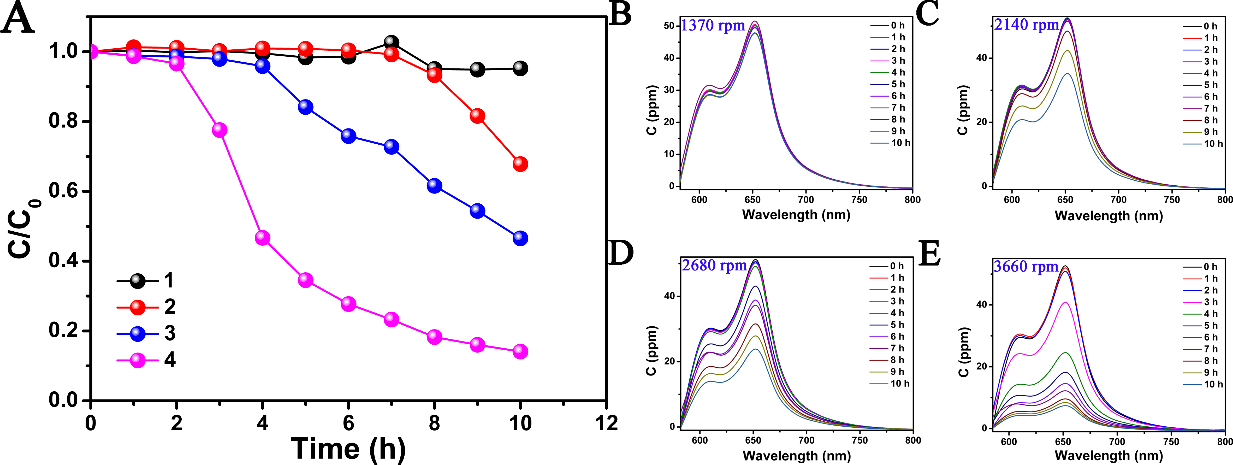


Figure S4. The C/C_0_ of U(VI) with time at different speeds (1:1370 rpm, 2:2140 rpm, 3:2680 rpm, 4:3660 rpm).


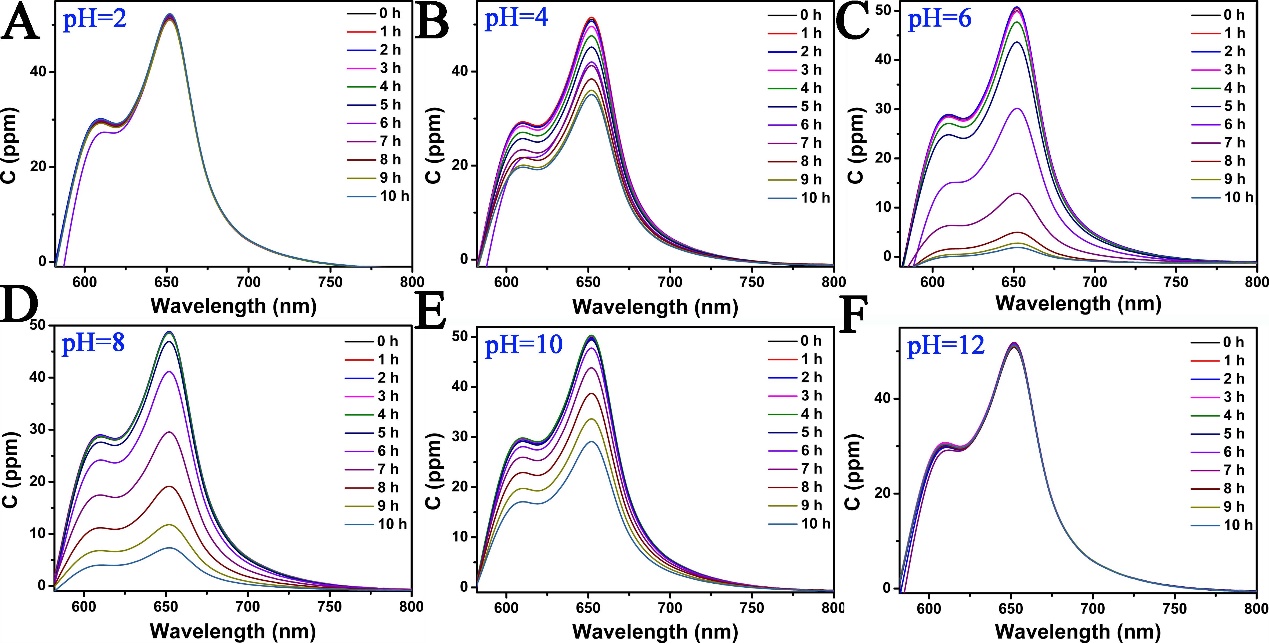


Figure S5. The U(VI) concentration in solution with time at different pH values.


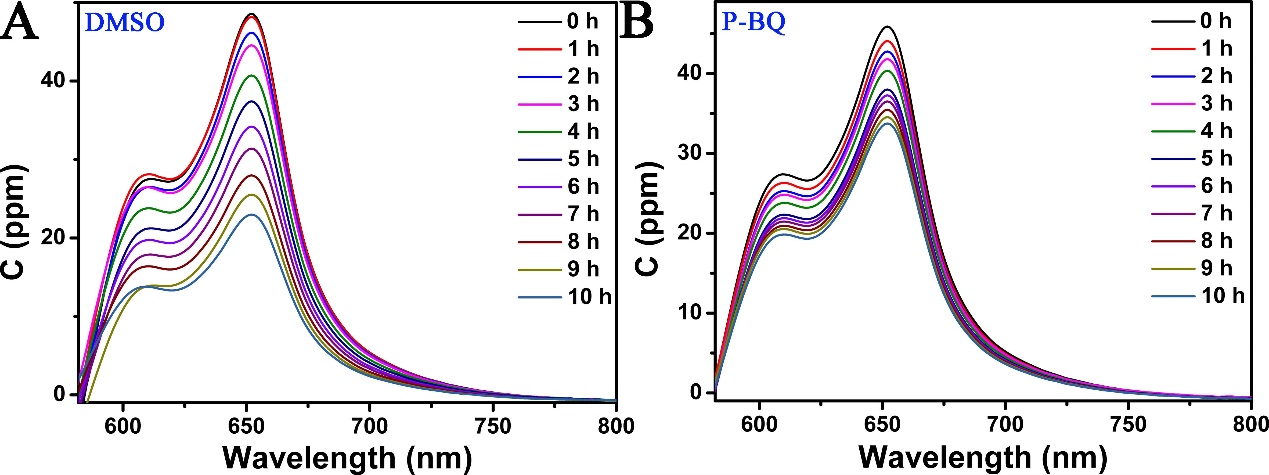


Figure S6. The catalysis experiment with different sacrificial agents.


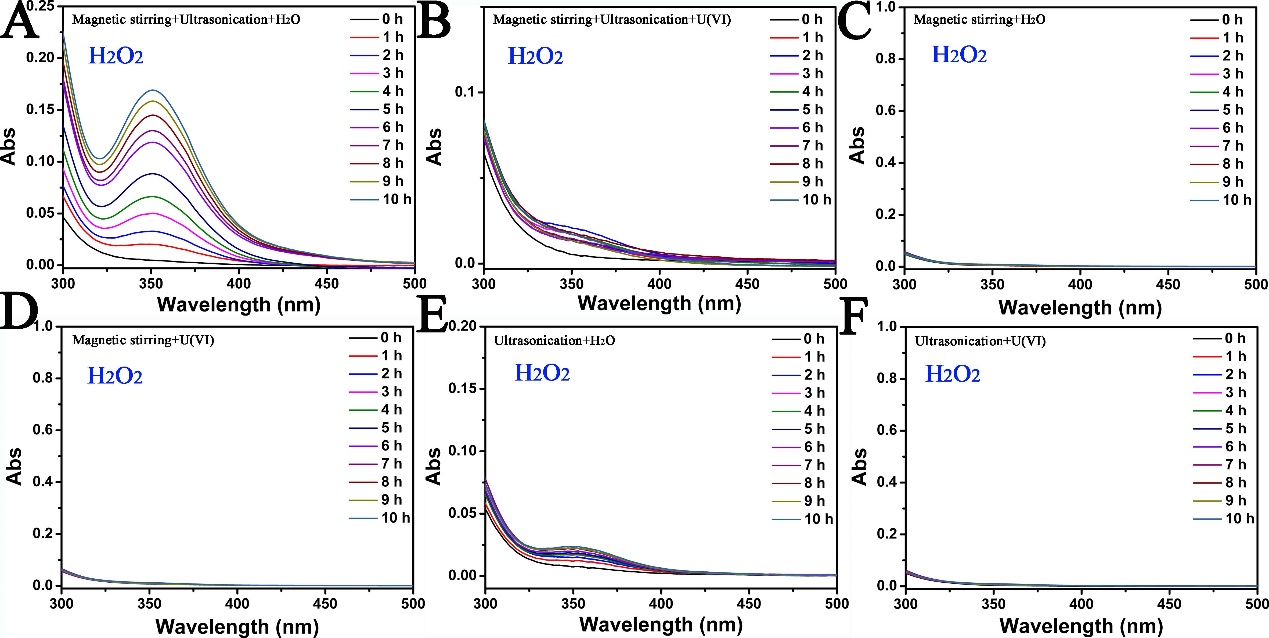


Figure S7. The curve of absorption with time for I_2_ under different conditions. A: The absorption of I_2_ in ultrapure water under magnetic stirring and ultrasonication; B: The absorption of I_2_ in U(VI) solution under magnetic stirring and ultrasonication; C: The absorption of I_2_ in ultrapure water under magnetic stirring; D: The absorption of I_2_ in U(VI) solution under magnetic stirring; E: The absorption of I_2_ in ultrapure water under ultrasonication; F: The absorption of I_2_ in U(VI) solution under ultrasonication.


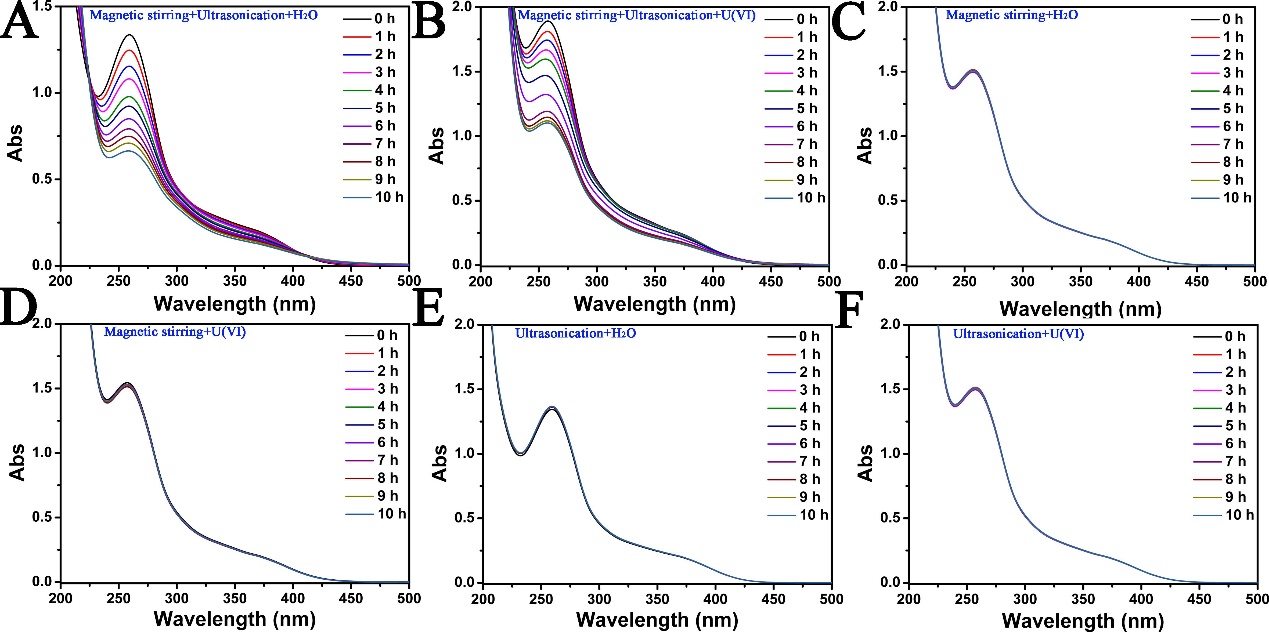


Figure S8. The curve of absorption for·O_2_^-^ with time under different conditions. A: The curve of absorption for·O_2_^-^ with time in ultrapure water under magnetic stirring and ultrasonication; B: The curve of absorption for·O_2_^-^ with time in U(VI) solution under magnetic stirring and ultrasonication; C: The curve of absorption for·O_2_^-^ with time in ultrapure water under magnetic stirring; D: The curve of absorption for·O_2_^-^ with time in U(VI) solution under magnetic stirring; E: The curve of absorption for·O_2_^-^ with time in ultrapure water under ultrasonication; F: The curve of absorption for·O_2_^-^ with time in U(VI) solution under ultrasonication.


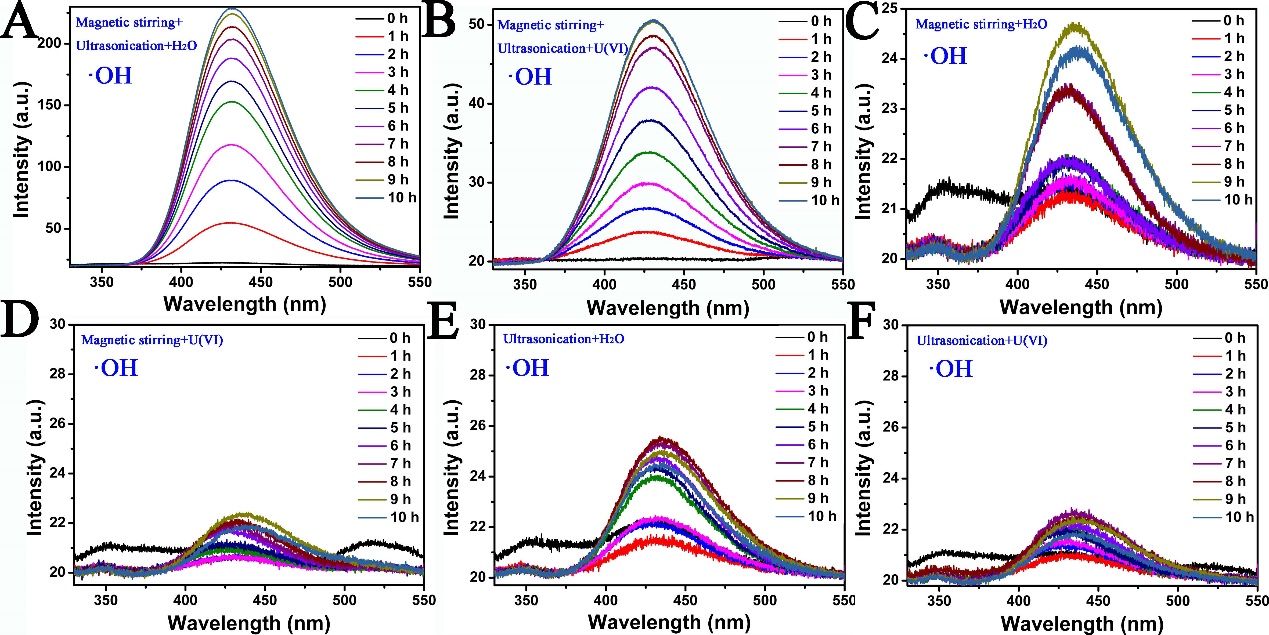


Figure S9. The curve of intensity for DHTA with time under different conditions. A: The curve of intensity for DHTA with time in ultrapure water under magnetic stirring and ultrasonication; B: The curve of intensity for DHTA with time in U(VI) solution under magnetic stirring and ultrasonication; C: The curve of intensity for DHTA with time in ultrapure water under magnetic stirring; D: The curve of intensity for DHTA with time in U(VI) solution under magnetic stirring; E: The curve of intensity for DHTA with time in ultrapure water under ultrasonication; F: The curve of intensity for DHTA with time in U(VI) solution under ultrasonication.


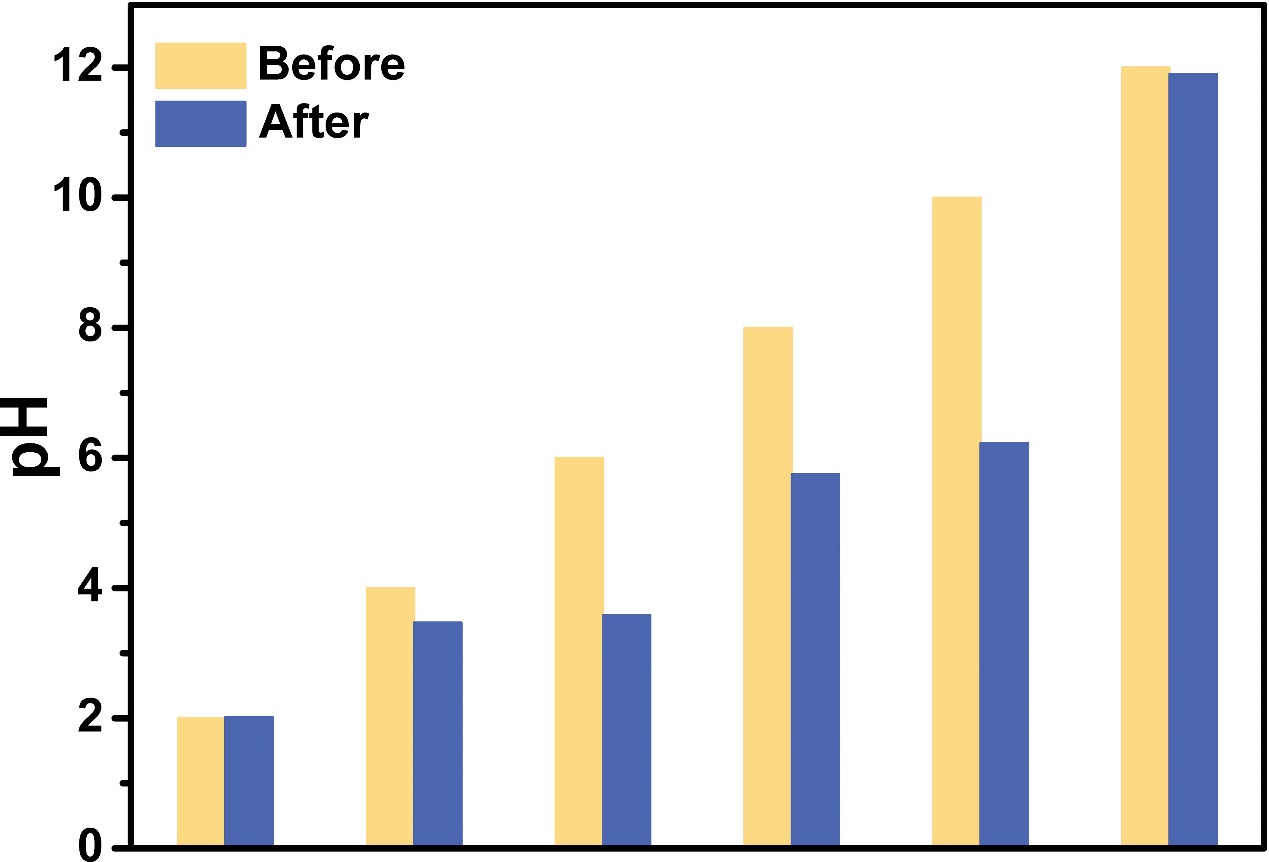


Figure S10. The pH of U(VI) solution before and after reaction.


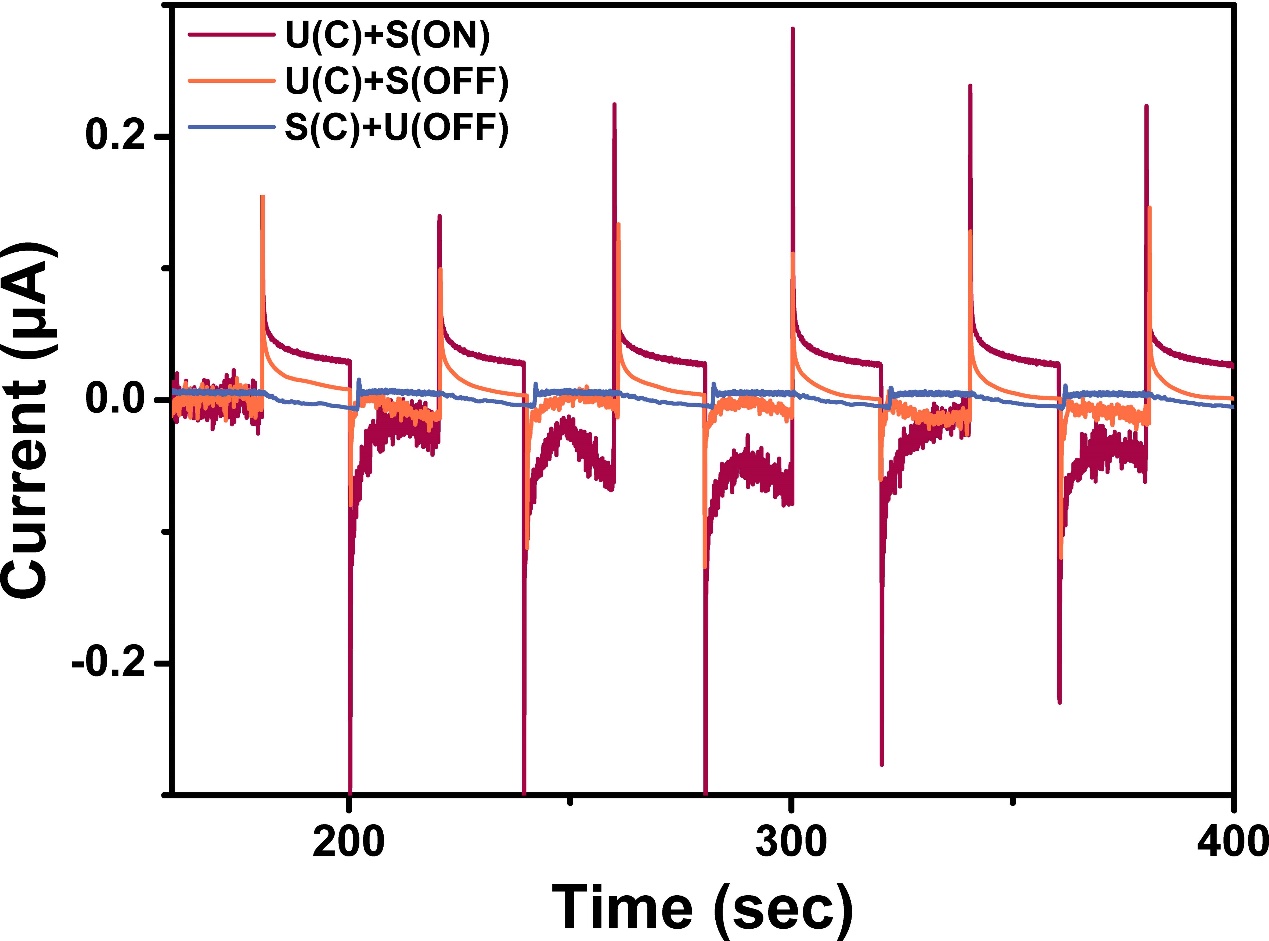


Figure S11. Current transient response in different conditions:"S" stands for stirring," U" stands for ultrasonic, "ON" means that the ultrasonic or stirring is always on, "OFF" means that it is always off, and "C" means that the current is periodically changed by controlling the switch.

It can be seen from Fig. S11 that when the ultrasound is always off, there is no significant change in the transient current by controlling the magnetic stirring on and off (the blue curves). However, a relatively obvious current response is observed by periodically switching on/off of ultrasound, especially in the presence of stirring. Most importantly, as the ultrasound and stirring both are turned on, the current is the biggest, indicating there generated the largest quantities of free radicals.


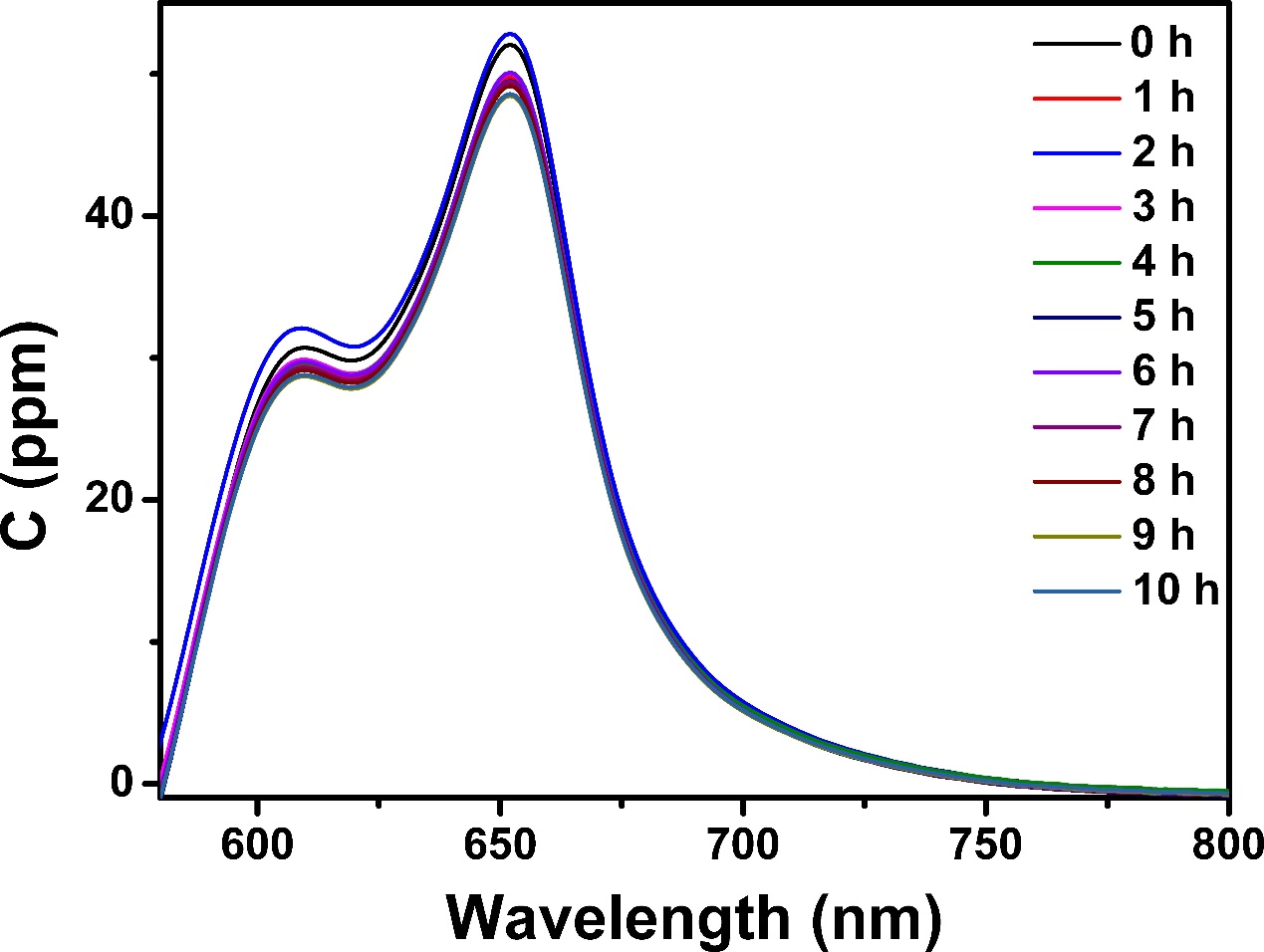


Figure S12. The curve of U(VI) concentration with time under mechanical stirring.


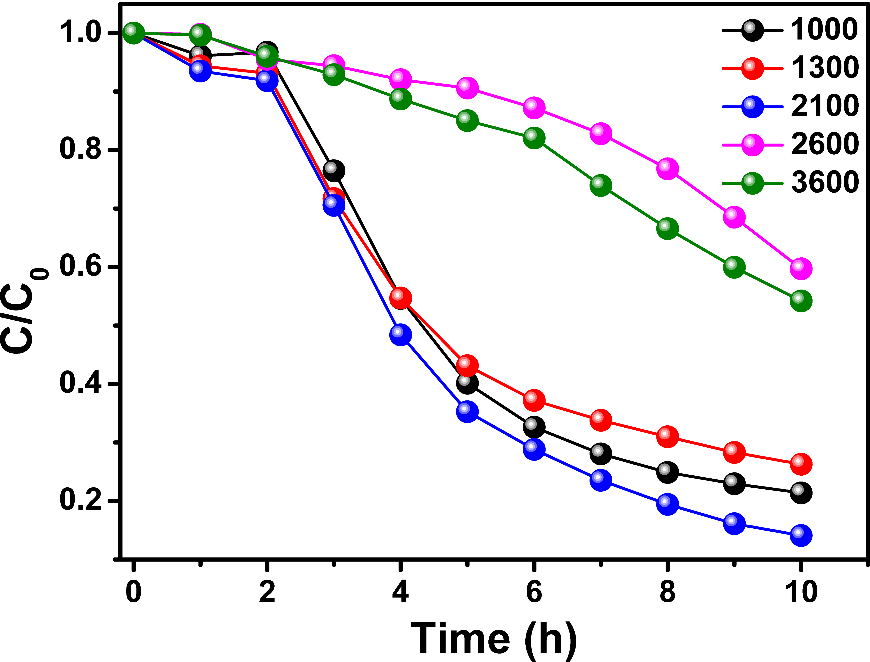


Figure S13. The C/C_0_ of U(VI) with time at different mechanical stirring speeds.


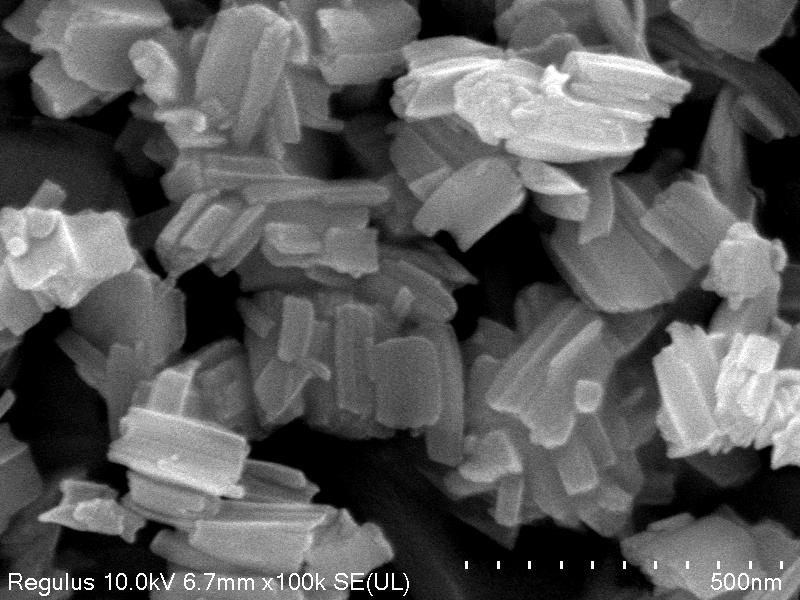


Figure S14. The SEM of the sample recovered by mechanical stirring.


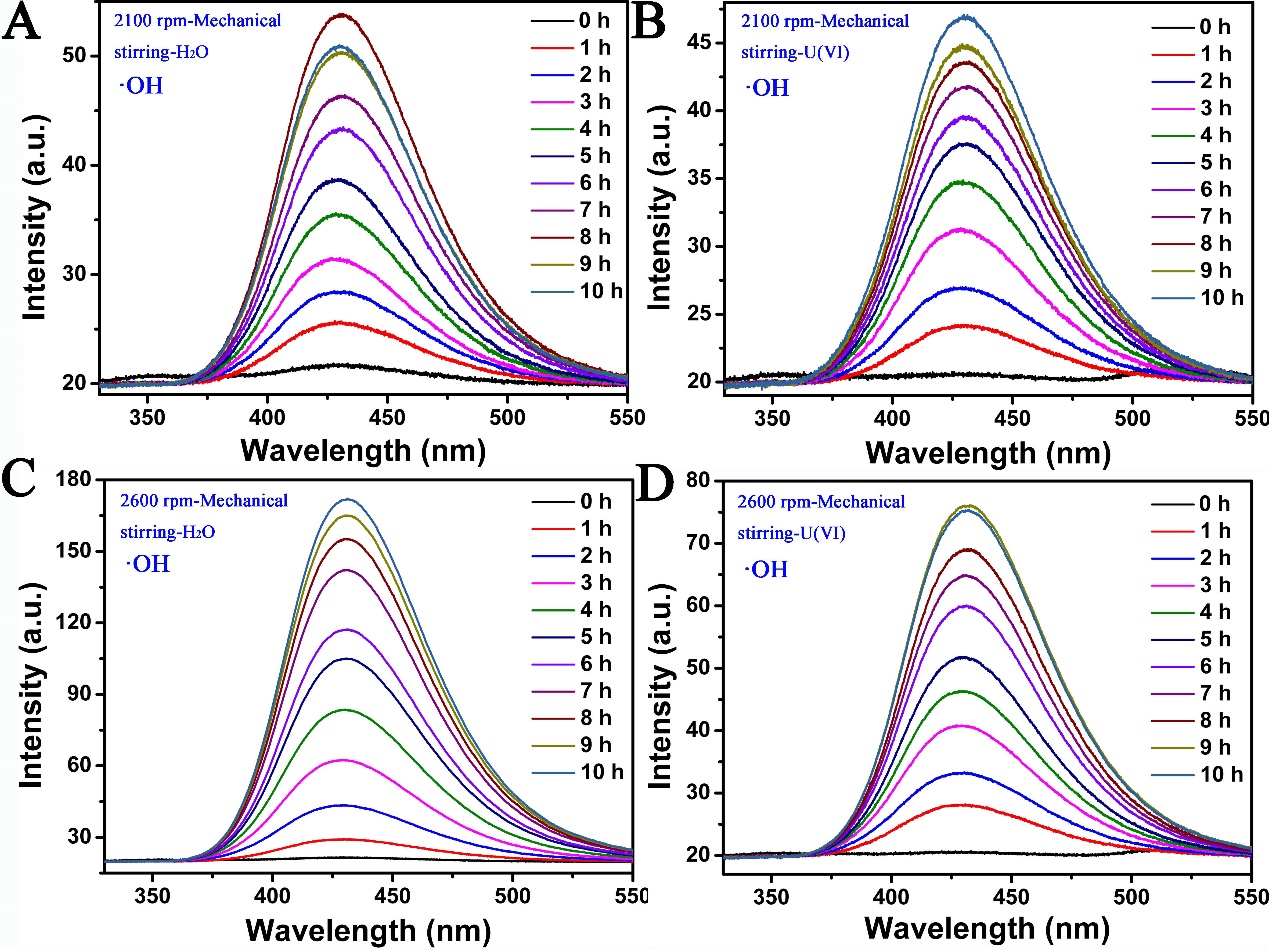


Figure S15. The curve of intensity for DHTA with time under different conditions. A: The curve of intensity for DHTA with time in ultrapure water under 2100 rpm mechanical stirring and ultrasonication; B: The curve of intensity for DHTA with time in U(VI) solution under 2100 rpm mechanical stirring and ultrasonication; C: The curve of intensity for DHTA with time in ultrapure water under 2600 rpm mechanical stirring and ultrasonication; D: The curve of intensity for DHTA with time in U(VI) solution under 2600 rpm mechanical stirring and ultrasonication.


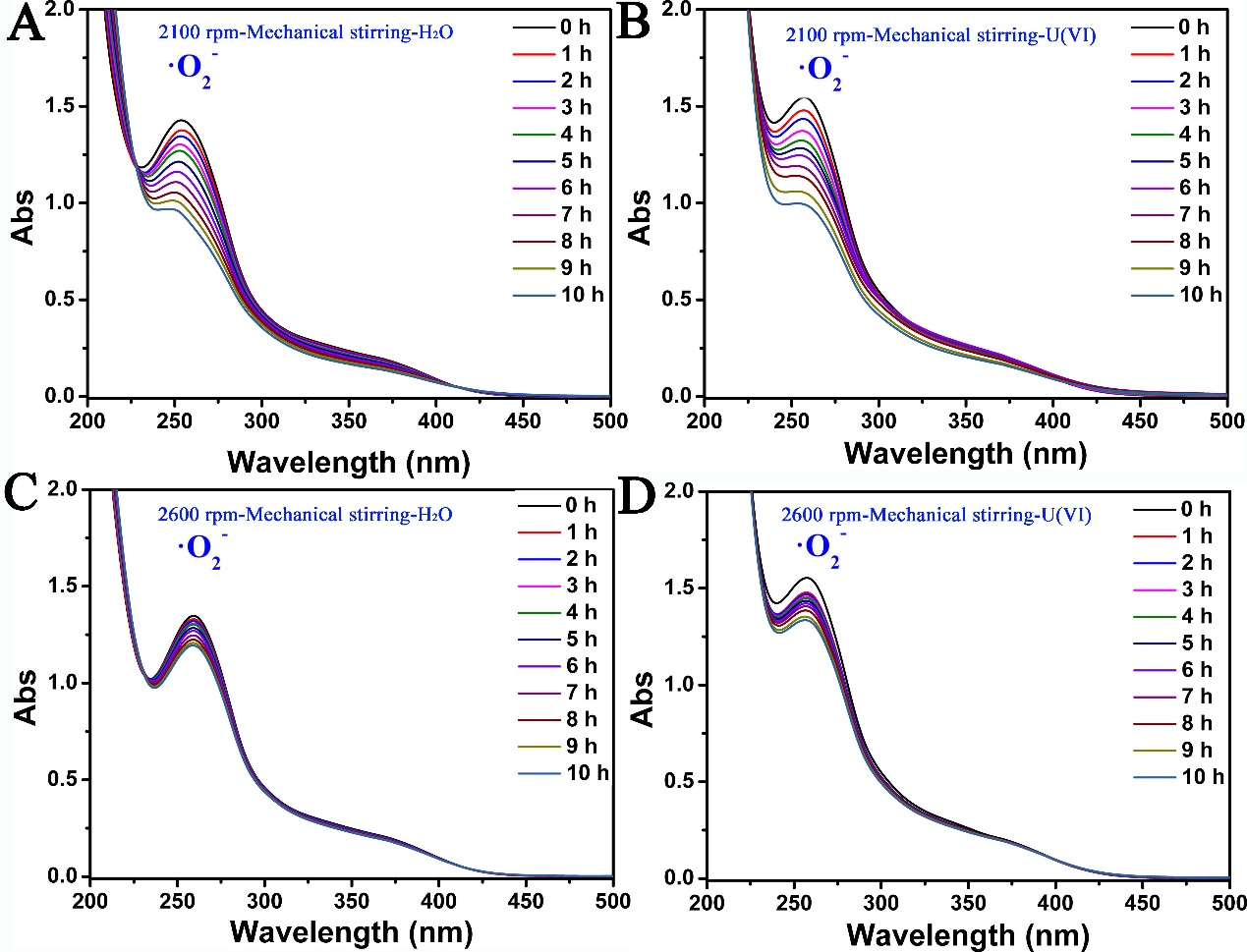


Figure S16. The curve of absorption for·O_2_^-^ with time under different conditions. A: The curve of absorption for·O_2_^-^ with time in ultrapure water under 2100 rpm mechanical stirring and ultrasonication; B: The curve of absorption for·O_2_^-^ with time in U(VI) solution under 2100 rpm mechanical stirring and ultrasonication; C: The curve of absorption for·O_2_^-^ with time in ultrapure water under 2600 rpm mechanical stirring and ultrasonication; D: The curve of absorption for·O_2_^-^ with time in U(VI) solution under 2600 rpm mechanical stirring and ultrasonication.


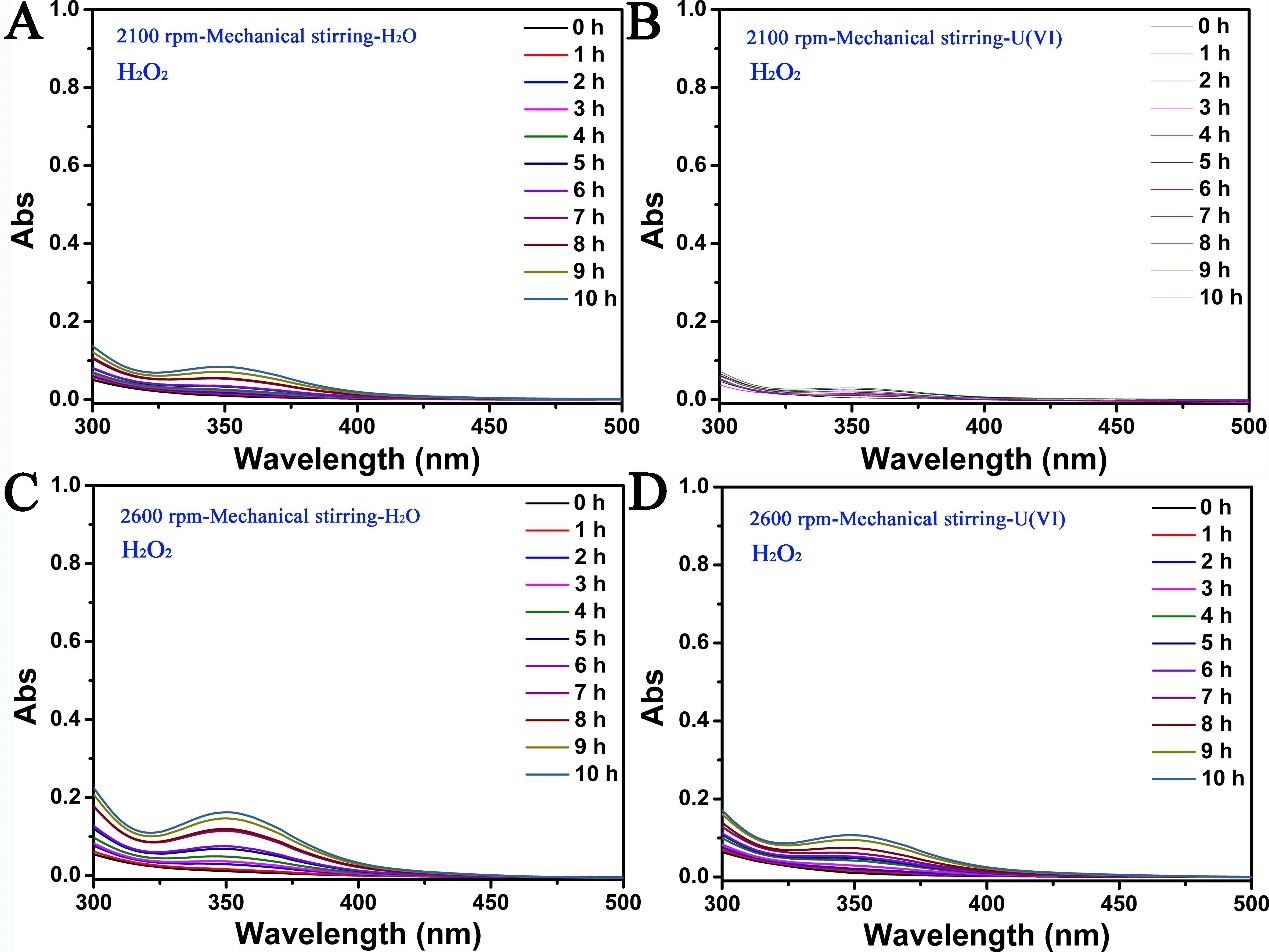


Figure S17. The curve of absorption for I_2_ with time under different conditions. A: The curve of absorption for I_2_ with time in ultrapure water under 2100 rpm mechanical stirring and ultrasonication; B: The curve of absorption for I_2_ in U(VI) solution under 2100 rpm mechanical stirring and ultrasonication; C: The curve of absorption for I_2_ in ultrapure water under 2600 rpm mechanical stirring and ultrasonication; D: The curve of absorption for I_2_ in U(VI) solution under 2600 rpm mechanical stirring and ultrasonication.


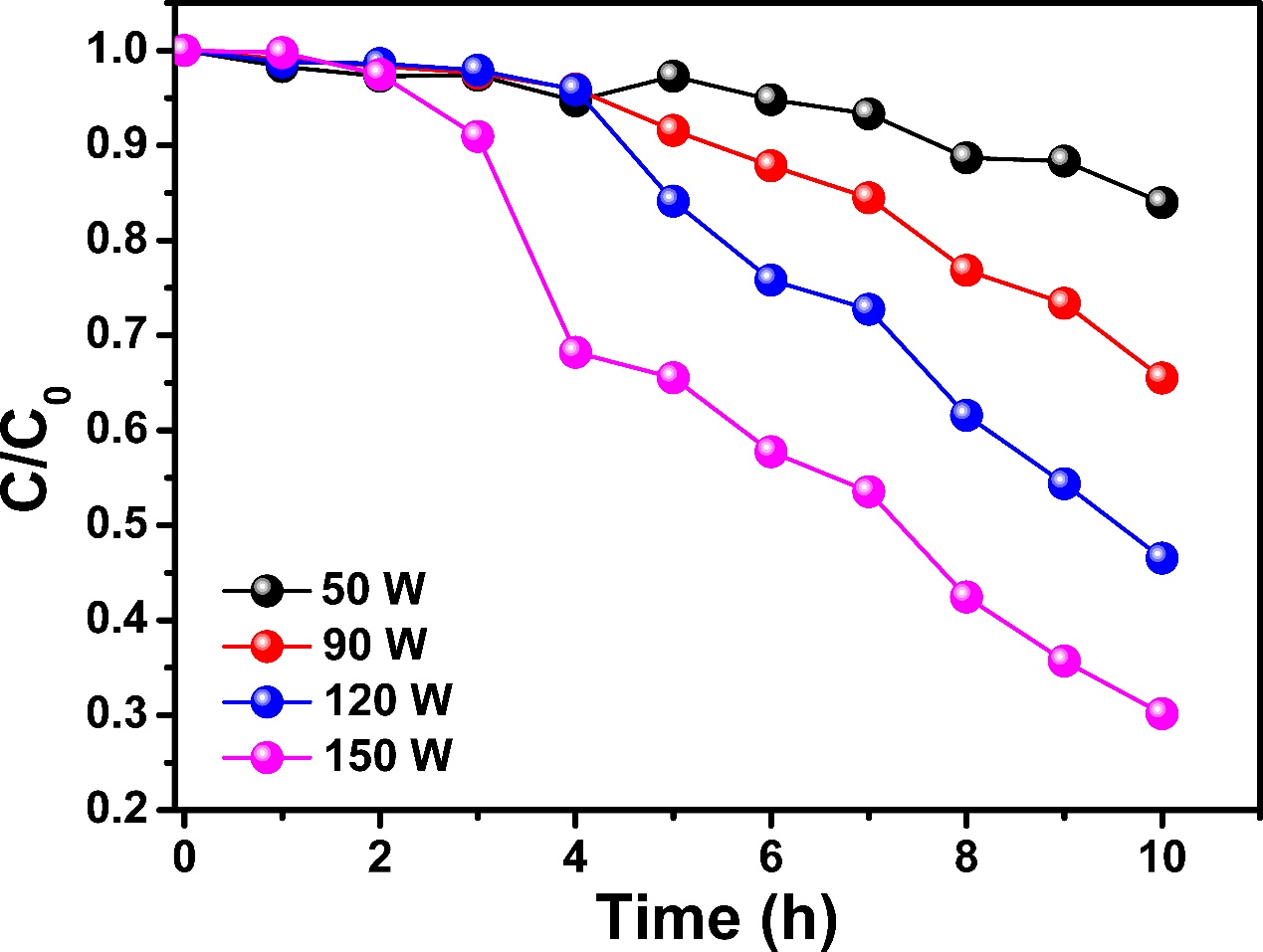


Figure S18. Curve of U(VI) concentration with time under different ultrasonic power.

Cavitation bubbles play a very important role in catalytic experiments. In fact, the generation of bubbles in the solution is related to the ultrasonic power. Fig. S18 shows the effect of ultrasonic power on the catalysis of U(VI). With the increase of ultrasonic power, the removal rate of U(VI) (10 h) increased from 16% (50 W) to 70% (150 W). The high power of the ultrasound, the large quantities of the bubbles. The number of cavitation bubbles is determined by their upper and lower radii. The lower limit radius ($R_{l}$) of the cavitation bubble can be described as:^[1]^

$R_{l}^{3}+\frac{2\sigma}{p_{0}}R_{l}^{2}-\frac{32\sigma^{3}}{27p_{0}\left( p_{0}-p_{A} \right)^{2}}=0$ (2)

where $\sigma$ is the surface tension of the liquid, $p_{0}$ is the static pressure of the liquid, $p_{A}$ is the sound pressure.

The upper limit radius ($R_{u}$) of the cavitation bubble can be described as:

$R_{u}=\frac{2}{\omega}(p_{A}-p_{0})\left( \frac{2}{\rho p_{A}} \right)^{\frac{1}{2}}{(1+\frac{2}{3p_{0}}\left( p_{A}-p_{0} \right))}^{\frac{1}{3}}$ (3)

where $\rho$ is the density of the solution, $\omega$ is the source frequency.

The relation between $p_{A}$ and $I_{A}$ is as follows:

$p_{A}=\sqrt{2\rho cI_{A}}$ (4)

where $c$ is the speed of sound, $I_{A}$ is for sound intensity.

The number of cavitation bubbles that can undergo cavitation within 1 s:

N=$\frac{1}{2}Af(\frac{1}{R_{1}^{2}}-\frac{1}{R_{u}^{2}})$ (5)

where A is an empirical constant, which is between 10^-10^~10^-15^ m^2^, $f$ is the frequency.

The yield per 1 s:

$V_{s}=\frac{V_{s}\left( t_{i} \right)}{2}Af(\frac{1}{R_{1}^{2}}-\frac{1}{R_{u}^{2}})$ (6)

It can be seen from the above formulas that the sound intensity is directly related to the upper and lower radii of the cavitation bubble. The upper and lower radii directly affect the number of cavitation bubbles and further affect the yield.


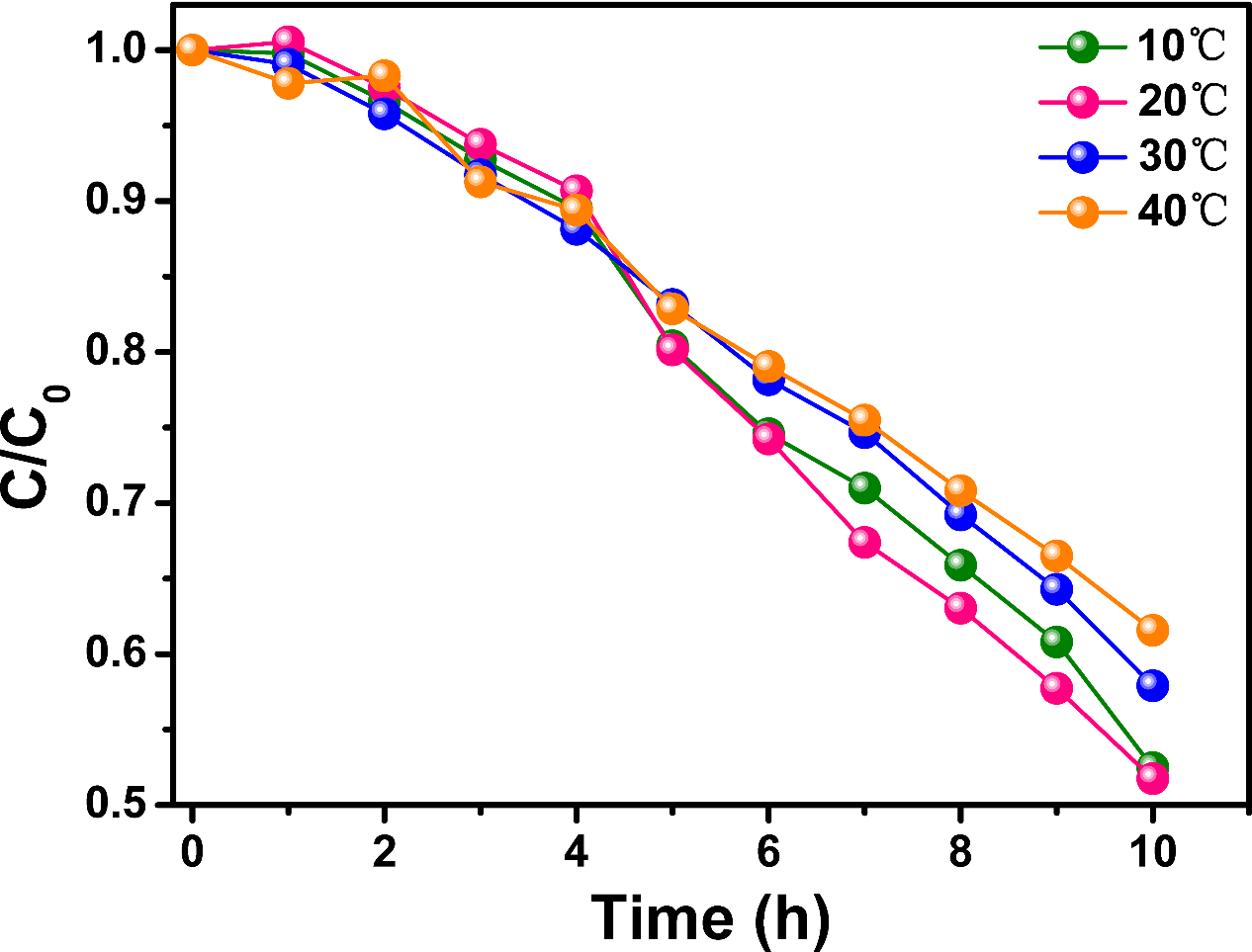


Figure S19. The C/C_0_ of U(VI) at different temperatures varies with time.

The effect of temperature on the removal/extraction of U(VI) by catalysis is carried out at four different temperatures (10 ℃, 20 ℃, 30 ℃, and 40 ℃). The increase in temperature makes the Brownian motion of molecules intense, which promotes the catalytic reaction^[2]^. It can be seen from Fig. S19 that compared with 10 ℃ and 20 ℃, the temperature increase plays a positive role in the catalytic removal of U(VI). As the temperature increases, the water's viscosity and surface tension decreases, while the vapor pressure increases. It can be seen from Equation 1 that the increase in temperature can indirectly lead to the decrease of the cavitation threshold, thus promoting the generation of cavitation bubbles.

$P_{c}=P_{0}-P_{v}+\frac{2}{3\surd3}\sqrt{\frac{{(\frac{2\sigma}{R_{0}})}^{3}}{P_{0}-P_{v}+\frac{2\sigma}{R_{0}}}}$ (7)

where $P_{0}$ is the hydrostatic pressure, $P_{v}$ is the vapor pressure, $\sigma$ is the surface tension of the liquid, $R_{0}$ is the initial radius of the cavitation core. However, the catalytic performance decreased from 48% (20 ℃) to 38% (40 ℃) as the temperature continued rising. This is because the high temperature can cause the escape of the dissolved gas from the solution, and the generation of H_2_O_2_ is reduced, thus reducing the reaction rate.


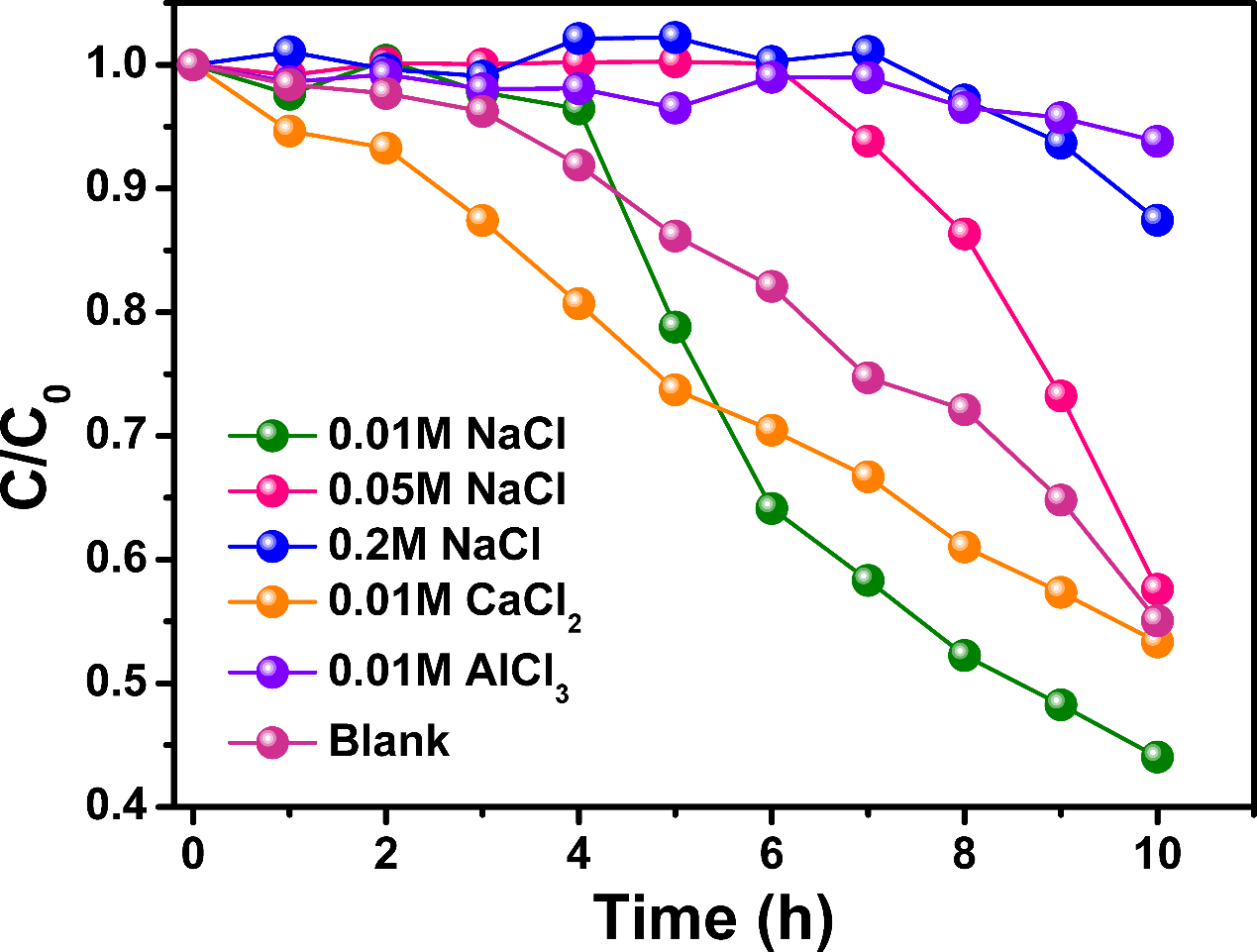


Figure S20. The C/C_0_ of U(VI) with time in the presence of different cations. Since the catalytic experiment is conducted at a U(VI) concentration of 50 ppm, all the "Blank" data mentioned in the paper are in the same group as the 50 ppm data in different concentration experiments for the convenience of comparison.

The effect of cations on the removal of U(VI) by catalysis is studied (Fig. S20). It can be clearly found from Fig. S20 that 0.01 M NaCl has little effect on catalysis, but with the increase of NaCl concentration (0.05 M NaCl and 0.2 M NaCl), it gradually shows inhibition on catalytic activity, and this inhibition becomes more intense with the increase of NaCl concentration. There are two possible causes for this result: (1) when the pH value is 4.4, U(VI) is positively charged, and the increase of the concentration of positively charged Na^+^ can compete with the positively charged UO_2_^2+^ and other active substances; (2) Cl^-^ can remove ·OH produced by advanced oxidation processes., the trapping experiment proves that the ·OH plays a positive role in the catalytic U(VI) process. Therefore, the inhibition effect of three different concentrations of NaCl on the removal of U(VI) follows the sequence of 0.2 M NaCl > 0.05 M NaCl > 0.01 M NaCl. In addition, the inhibition effect of cations on the removal of U(VI) by catalysis is gradually strengthened with the increase of the valence state and ionic radius of cations. This view can be seen from the comparison of NaCl, CaCl_2_ and AlCl_3_ with the same concentration of 0.01 M in Fig. S20.


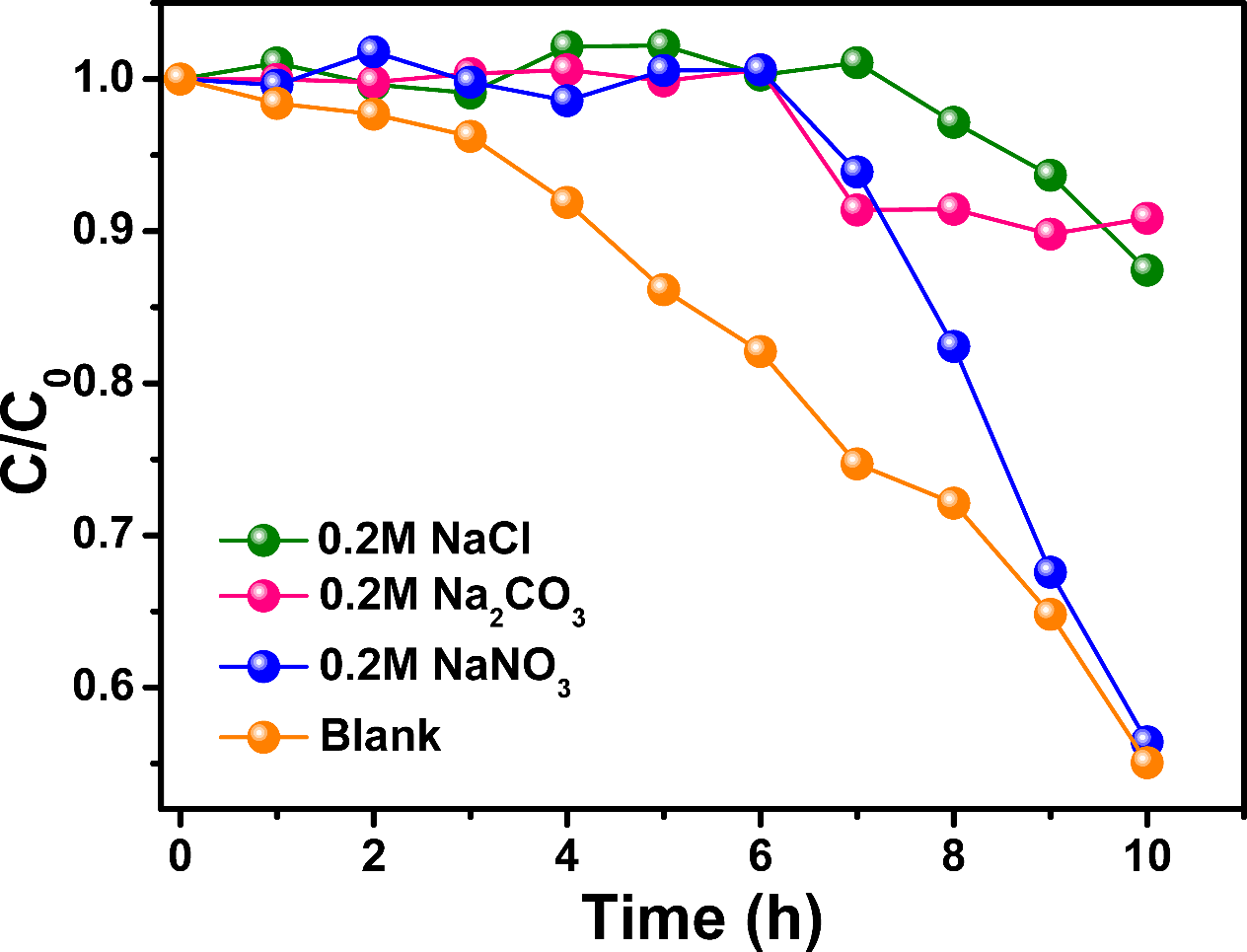


Figure S21. The C/C_0_ of U(VI) with time in the presence of different anions. Since the catalytic experiment is conducted at a U(VI) concentration of 50 ppm, all the "Blank" data mentioned in the paper are in the same group as the 50 ppm data in different concentration experiments for the convenience of comparison.

The effect of anions on catalytic performance has also been investigated (Fig. S21). CO_3_^2-^ can form a complex with U(VI), and this complex is highly water-soluble, which is not conducive to the removal of U(VI). It can also be seen from Fig. S21 that the presence of CO_3_^2-^ has a very strong inhibitory effect on uranyl in catalysis. It may also be because the addition of CO_3_^2-^ hydrolyzes to produce HCO_3_^-^ (Eq. (8)), and HCO_3_^-^ that can consume H_2_O_2_ and thus inhibit the extraction of solid uranium, as shown in Eq. (9)^[3]^. For NaNO_3_, the main reason that NO_3_^-^ inhibits catalytic U(VI) is that NO_3_^-^ can act as a strong collector, similar to Cl^-^ mentioned above.

CO_3_^2-^ + H_2_O = HCO_3_^-^ +OH^-^ (8)

HCO_3_^-^ + H_2_O_2_ = HCO_4_^-^ + H_2_O (9)

# Supplementary Table

Table S1. Free energy is calculated by DFT.

| ROS | Free Energy (eV) |
| --- | --- |
| ·OH | 4.93 |
| ·O_2_^-^ | 0.66 |
| H_2_O_2_ | 1.67 |

# References

[1] Y. Liu, Z. Zhao, Q. Zhao, *JOURNAL OF CHEMICAL INDUSTRY ENGINEERING-CHINA-* **2002**, *53*, 1206-1208.

[2] a) S. Zhang, H. Liu, F. Gao, M. Fang, Y. Zhang, Y. Cai, K. Li, M. Kong, X. Tan, *Journal of Alloys Compounds* **2022**, *900*, 163492; b) C.-C. Hsueh, B.-Y. Chen, *Journal of Hazardous Materials* **2007**, *141*, 842-849.

[3] R. Drago, K. Frank, Y. C. Yang, G. Wagner, in *Proceedings of the 1997 ERDEC Scientific Conference on Chemical and Biological Defense Research, US Army Edgewood Research, Development, and Engineering Center, Aberdeen Proving Ground, MD*, **1998**.
